# Homeostatic Interplay between Bacterial Cell-Cell Signaling and Iron in Virulence

**DOI:** 10.1371/journal.ppat.1000810

**Published:** 2010-03-12

**Authors:** Ronen Hazan, Jianxin He, Gaoping Xiao, Valérie Dekimpe, Yiorgos Apidianakis, Biliana Lesic, Christos Astrakas, Eric Déziel, François Lépine, Laurence G. Rahme

**Affiliations:** 1 Department of Surgery, Harvard Medical School and Massachusetts General Hospital, Boston, Massachusetts, United States of America; 2 Department of Microbiology and Molecular Genetics, Harvard Medical School, Boston, Massachusetts, United States of America; 3 INRS-Institut Armand-Frappier, Laval, Québec, Canada; The University of Texas at Austin, United States of America

## Abstract

Pathogenic bacteria use interconnected multi-layered regulatory networks, such as quorum sensing (QS) networks to sense and respond to environmental cues and external and internal bacterial cell signals, and thereby adapt to and exploit target hosts. Despite the many advances that have been made in understanding QS regulation, little is known regarding how these inputs are integrated and processed in the context of multi-layered QS regulatory networks. Here we report the examination of the *Pseudomonas aeruginosa* QS 4-hydroxy-2-alkylquinolines (HAQs) MvfR regulatory network and determination of its interaction with the QS acyl-homoserine-lactone (AHL) RhlR network. The aim of this work was to elucidate paradigmatically the complex relationships between multi-layered regulatory QS circuitries, their signaling molecules, and the environmental cues to which they respond. Our findings revealed positive and negative homeostatic regulatory loops that fine-tune the MvfR regulon via a multi-layered dependent homeostatic regulation of the cell-cell signaling molecules PQS and HHQ, and interplay between these molecules and iron. We discovered that the MvfR regulon component PqsE is a key mediator in orchestrating this homeostatic regulation, and in establishing a connection to the QS *rhlR* system in cooperation with RhlR. Our results show that *P. aeruginosa* modulates the intensity of its virulence response, at least in part, through this multi-layered interplay. Our findings underscore the importance of the homeostatic interplay that balances competition within and between QS systems via cell-cell signaling molecules and environmental cues in the control of virulence gene expression. Elucidation of the fine-tuning of this complex relationship offers novel insights into the regulation of these systems and may inform strategies designed to limit infections caused by *P. aeruginosa* and related human pathogens.

## Author Summary

Bacterial cells can communicate with one another about their surrounding environment. This information can be in the form of small self-secreted molecules acting as signals to activate or inhibit the expression of genes. *Pseudomonas aeruginosa* is an environmental bacterium that infects diverse organisms from plants to humans. Our results show that this pathogen uses two highly sensitive networks, namely MvfR and LasR/RhlR pathways, to modulate its virulence functions by titrating the concentration of the small molecules HHQ and PQS in a manner that depends upon the presence or absence of iron. Via negative and positive feedback loops, this bacterium processes the signaled information to regulate its virulence functions and homeostatically balance the production of the small molecules required for the activation of the MvfR virulence network. Our study sheds light on paradigmatic complex networks that maintain a homeostatic bacterial virulence response.

## Introduction

Microbes translate environmental cues to coordinate and modulate gene expression such that they can adapt to different niches and overcome hostile environments. Adaptation and coordination of gene expression is particularly important for pathogenic microorganisms that need to colonize dynamic host environments since their ability to sense and respond to host environmental cues is critical for their survival. In bacteria, modulation and coordination of gene expression are also influenced by population density via the regulated production of small molecules that serve as intricate signals impacting the expression of virulence factor genes. Many studies have addressed the role of quorum sensing (QS) communication networks in virulence where by diffusible intercellular auto-inducers factor and environmental signals bacterial cultures mediate pathogenicity by coordinating the expression of a large array of genes [1],[2]. Nevertheless, less is known regarding how environmental cues are translated in the context of QS signaling and how environmental cues and QS are integrated to promote the ability of a pathogen to survive and colonize particular niches within their host environments. The processing and integration of environmental inputs in QS becomes even more complex when a pathogen is able to occupy more than one niche.

*Pseudomonas aeruginosa* is a ubiquitous and an extremely versatile Gram-negative bacterium with an astounding ability to survive in many different environments and to infect multiple hosts ranging from amoebas to humans [3]. This pathogen has an extensively studied complex QS communication network that facilitates cross-talk between organisms and impacts many *P. aeruginosa* group-related behaviors including virulence [4],[5],[6],[7],[8],[and 9]. There are at least three known QS systems in *P. aeruginosa*: two are dependent on the acyl-homoserine-lactone (AHL) QS transcription factors LasR and RhlR [10] and a third is dependent on the 4-hydroxy-2-alkylquinolines (HAQs) LysR-type transcription factor MvfR [11],[12]. MvfR activation is mediated by the cell-cell signaling molecules 4-hydroxy-2-heptylquinoline (HHQ) and 3,4-dihydroxy-2-heptylquinoline (PQS), and leads to the positive regulation of many virulence-related factors, a large number of which are also controlled by the QS signal acyl-homoserine-lactone (AHL)-mediated RhlR and LasR circuitry.

The MvfR pathway is a critical virulence component essential for the full virulence of *P. aeruginosa* in multiple hosts [13],[14],[15] and is connected to LasR and RhlR by: (i) the dependence of *mvfR* expression at the early growth stages as a result of positive control by LasR [16], (ii) the conversion of HHQ into PQS controlled by PqsH [17],[18] whose expression is mediated by LasR [19],[20], and (iii) the negative effects of RhlR on the *pqs* operon [16],[21], which is responsible for the synthesis of all HAQs [11],[14],[19],[22],[23] including the MvfR ligands HHQ and PQS [12],[17],[21].

The QS regulons MvfR, LasR and RhlR respond not only to QS signal molecules but also to environmental signals [24], including host factors [25],[26],[27],[28] and other environmental cues such as phosphate [29], magnesium [30] and iron [31],[32],[33],[34],[35]. Iron acquisition is controlled by a large set of *P. aeruginosa* genes activated in response to iron starvation [36],[37],[38], including two siderophore complexes, pyoverdine and pyochelin [39],[40], and several ferric uptake regulators, among them are the general iron uptake regulator Fur, Fur-regulated pyoverdine siderophore-specific extracytoplasmic sigma factor PvdS, several ECF sigma factors, and the AraC regulator PchR, which regulates pyochelin uptake [40]. In low iron conditions, PvdS binds to iron-starvation (IS) boxes to induce the transcription of many genes involved in the iron starvation response [41]. The intricate relationship between QS and iron is exemplified by a series of findings demonstrating that iron starvation induced QS systems [26],[32],[34] and that the QS regulators MvfR [11], LasR/RhlR [42] and VqsR [31],[43],[44] were found to be responsible for the induction of many iron response genes. Moreover, MvfR contains an IS box in its promoter [36], and PQS production is positively-affected by two Fur-regulated small RNAs, Prrf 1 and 2 [45]. Adding to the complexity of how environmental cues such as iron levels affect QS and how iron is integrated into QS to modulate virulence gene expression is the ability of PQS to bind iron [46], to act as an iron trap molecule [47], and to form a toxic complex against the host [48].

MvfR activation by HHQ and PQS leads to the upregulation of the anthranilic acid (AA)- biosynthetic encoding genes *phnAB*, and *pqsA-E* operon [11],[12],[14] that have a conserved genomic organization in *P. aeruginosa* and in HAQs-producing *Burkholderia* species [49], to produce more HAQs leading to the upregulation of the MvfR-regulon in a positive feedback loop. Although the fifth gene of the *pqs* operon *pqsE* (PA14_51380), which encodes a predicted GloB, Zn-dependent hydrolase [50] and member of the metallo-beta-lactamase super family (Pfam PF00753), is not required for HAQ synthesis [12],[19], it is co-regulated together with the *pqsA-D* genes. We have shown that PqsE is essential for complete *P. aeruginosa* virulence in mice because it controls the expression of a number of MvfR regulon-dependent genes [11]. Although PqsE was previously implicated as the PQS response gene [19],[20], it was recently shown to act independently of MvfR and PQS [51]. Thus, the PqsE functions associated with the integration and translation of the QS cell-cell signals has yet to be resolved.

Here we examine the interplay between environmental cues and cell-cell signaling molecules and assess how they are integrated in the modulation of MvfR regulon gene expression. To elucidate the QS multi-layered regulation, we also examine the functional dependency of the MvfR regulon components, especially PqsE, and PQS and HHQ, on the Rhl regulon. The findings presented offer new insights into the highly complex *P. aeruginosa* virulence-associated regulatory loops that may aid in understanding and controlling its pathogenicity.

## Results

### Dissection of the QS MvfR regulon reveals a key component functioning independently of the cell-cell signaling molecules PQS and HHQ

**Figure 1:**
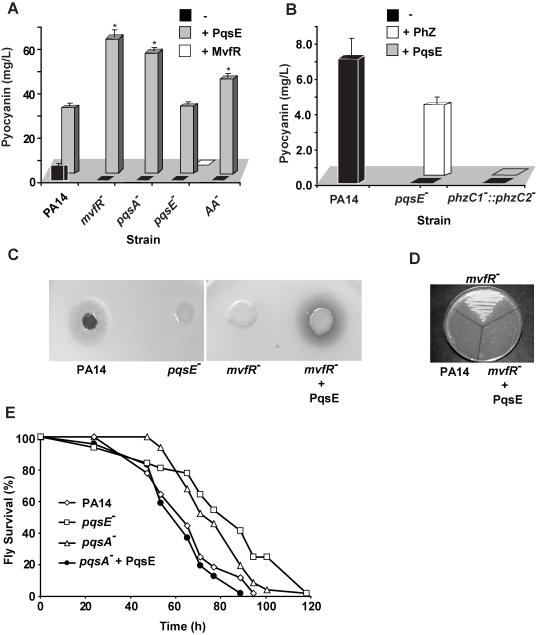
PqsE, a key mediator of the MvfR regulon activation, functions independently of AA and its derivatives. (A and B) Pyocyanin production was measured from PA14 and mutants with and without constitutive expression of PqsE or MvfR as a consequence of the presence of pDN19*pqsE* or pDN18*mvfR* plasmids, respectively. (A) AA^−^ is a triple mutant with non-functional *phnAB*, *trpE* and *kynBU* that does not produce anthranilate. Production of pyocyanin (+ Phz) was achieved by co-culturing two sets of cells one constitutively expressing *phzA2-G2*, and the other *phzM* and *phzS* genes encoding the phenazines and pyocyanin biosynthetic genes respectively. Asterisks in A show strains harboring the plasmid pDN19*pqsE* that are significantly different (P value <0.01) from PA14 harboring that plasmid. (C–D) PqsE is essential for the virulence of *P. aeruginosa* against *Cryptococcus neoformans* independently of HAQs. PqsE was constitutively expressed in *mvfR^−^* mutant cells. An empty vector served as a control (−). (C) 1 µL of bacterial culture was spotted onto YPD top-agar where yeast cells were plated. Yeast killing zones were formed only around the PA14 and mutant cells expressing PqsE. (D) The death of yeast cells within the killing zone was demonstrated by assessing their viability on YPD plates. (E) PqsE causes fly mortality in absence of HAQs. Survival kinetics of *Drosophila melanogaster* was assessed using a fly feeding assay. The survival kinetics of *pqsA* ^−^ and *pqsE* ^−^ infected flies was significant different (P value <0.005) form that of PA14-infected flies. However, the kinetics of *pqsA* ^−^ + PqsE- infected flies did not differ significantly from that of the PA14-infected flies(P value = 0.27).

To elucidate how multi-layered regulatory networks sense and respond to external and internal cell signals to modulate gene expression, we studied the role of MvfR pathway components in integrating and translating signals from PQS and HHQ in the activation of the MvfR regulon genes. To this end, we measured pyocyanin production as an index. This secreted *P. aeruginosa* phenazine was chosen since its production is dependent on the MvfR pathway components, including the cell-cell signaling molecules, PQS and HHQ, and their corresponding biosynthetic enzymes PqsA-D, their AA precursor, PqsE, and on its Phz biosynthetic operons (Figure 1A and [11]). Here we found that overexpression of PqsE under a constitutive promoter (pDN19*pqsE*) in *pqsA* ^−^ and *mvfR* ^−^ mutant cells not producing HAQs restored pyocyanin production (Figure 1A). In contrast, overexpression of *mvfR* under a constitutive promoter in a *pqsE^−^* background did not restore pyocyanin production (Figure 1A) even when HHQ, PQS, or PA14 cell-free supernatants were added (data not shown). These results highlight the crucial role of PqsE in the regulation of MvfR regulon-dependent factors and demonstrate that PqsE possesses activation properties that are independent of HAQ-mediated signals (Table S1). To assess PqsE mode of action on pyocyanin production, we co-cultured *pqsE^−^* cells constitutively expressing the phenazine biosynthetic operon *phzA2-G2* with *pqsE^−^* cells harboring the *phzM and phzS* genes essential to pyocyanin synthesis [52] and assessed pyocyanin production. As shown in Figure 1B, approximately 60% of the pyocyanin production was restored, indicating that PqsE participated in pyocyanin production regulation rather than in its synthesis.

Second, we tested whether the precursor of all HAQs, AA was required for PqsE function instead. To this end we used a triple mutant strain deficient in *phnAB*, *trpE* and *kynBU* (AA^−^ mutant) unable to produce any AA since all three AA synthesis pathways were knocked out [53]. Expression of PqsE in this triple mutant also resulted in high levels of pyocyanin production (Figure 1A) corroborating with the above results and demonstrating that PqsE function did not require AA or any of its derivatives to promote production of the MvfR regulon-dependent factor pyocyanin.

Third, since PqsE controlled the regulation of one of the key MvfR-regulated factors, pyocyanin, we sought to define the impact of this factor in the regulation of all MvfR-dependent virulence genes. We carried out whole genome expression studies and compared the expression profiles of a *pqsE* ^−^ mutant to those of the PA14 parental strain, an *mvfR* ^−^ mutant and to those of PA14 and an *mvfR* ^−^ over-expressing *pqsE* strain (NCBI GEO, accession number #GSE17147). These results showed that PqsE profoundly affected the expression of 90% of the MvfR-regulated genes, including at least thirty-six known and predicted transcription factors (Tables S1B and S2). Of the PqsE-dependent genes, 241 were found to be negatively regulated and 384 positively regulated by PqsE (Table S1). At least 75 positively-regulated genes encoded for putative or known virulence factors (Table S1) [11],[42]. Importantly, included among the positively-regulated virulence transcriptional factors was the QS AHL regulator *rhlR* [38] and iron response genes, including the iron starvation sigma factor *pvdS* and genes involved in the synthesis of the siderophore complex pyochelin (Table S3A).

To confirm that PqsE overexpression also restores virulence functions apart from restoring their expression independently of the signaling molecules PQS and HHQ, we used two assays. The first is based on the observation that virulent *P. aeruginosa* strains; including PA14 kill yeast [54],[55],[56]; and the second is based on that *P. aeruginosa* can infect and kill *Drosophila melanogaster* [57],[58],[59], and that *mvfR* mutant cells exhibit attenuated virulence in flies [57]. As illustrated in Figure 1C–D, a zone of yeast growth inhibition was observed around PA14, but not around the *mvfR* ^−^, or *pqsE^−^* mutants following plating of *C. neoformans* KN99α 5 mm from the bacterial colony on a YPD plate (Figure 1D). The killing zone was restored following PqsE overexpression in *mvfR* ^−^ backgrounds (Figure 1C–D). In agreement flies infected with *pqsA* ^−^ or *pqsE* ^−^ mutants cells exhibited significant delayed in mortality compared to that caused by the WT or the pqsA^−^ cells expressing pqsE (Figure 1E) demonstrating again that PqsE is crucial for *P. aeruginosa* pathogenicity and independent of PQS and HHQ.

### MvfR-dependent gene regulation relies on the functional cooperation between RhlR and PqsE

**Figure 2:**
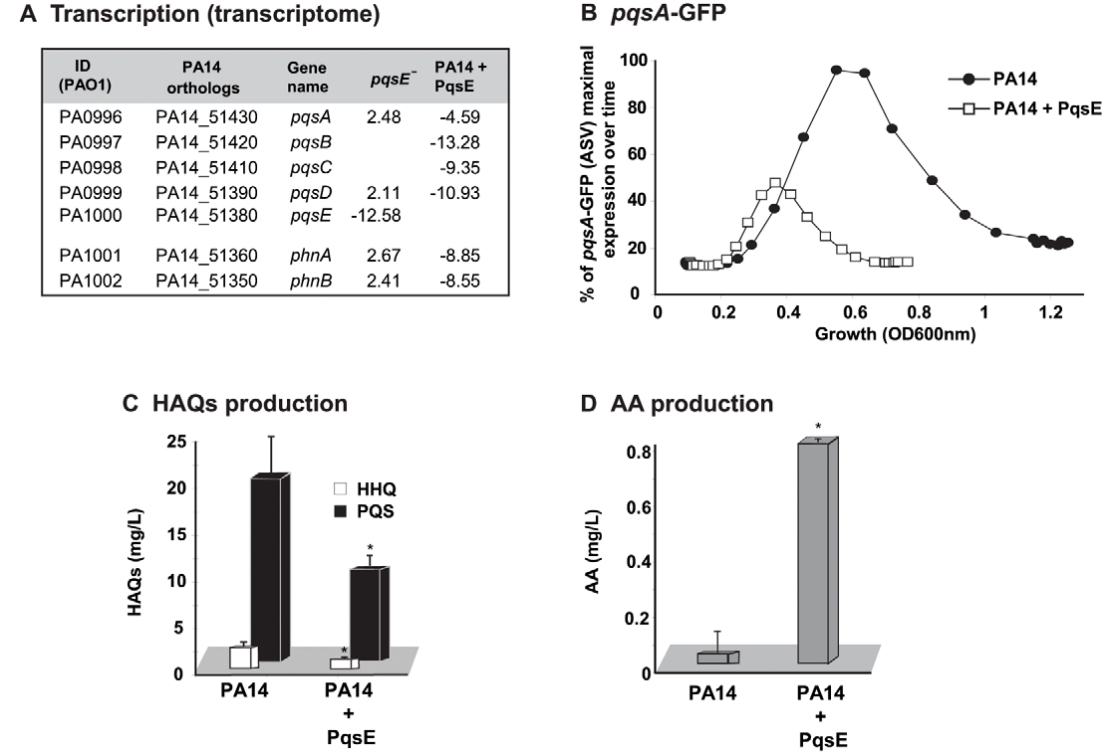
The homeostatic regulation of the signaling molecules HHQ and PQS is orchestrated by PqsE. Effect of PqsE on *pqs* operon gene expression, and production of HAQs and AA. (A) Fold change in expression of *phn* and *pqs* operons in *pqsE* ^−^ mutant and PA14 constitutively expressing PqsE versus PA14. (B) GFP intensity derived from a *pqsA*-GFP(ASV) reporter fusion; (C) HAQs and (D) AA levels as assessed by LC-MS. t-tests (p = 0.001 for HHQ and p = 0.004 for PQS) showed that the difference between PA14 and PA14+PqsE is statistically significant.

Comparison of the *pqsE* transcriptome (Table S1) to *lasR*/*rhlR* [42] revealed that almost half (46%) of the genes regulated by LasR/RhlR were also regulated by PqsE (Figure S3A) indicating a relationship between AHL- and MvfR-mediated QS regulons. This relationship is also extended to the negative effects that both components have on the transcription of the *pqs* operon ([16] and Table S1 and Figure 2A). A green fluorescent protein (GFP) reporter gene [32] fused to the *pqs* operon promoter (Figures 2B), quantitative PCR analysis (Figure S2D) and quantification of HHQ and PQS levels (Figure 2C) further validated the above finding. Moreover, in agreement, Figure 2D shows that HAQ synthesis down-regulation paralleled the accumulation of AA (HAQ precursor) followed by an increase in *antABC* gene expression that encodes enzymes for AA degradation (Table S1).

**Figure 3:**
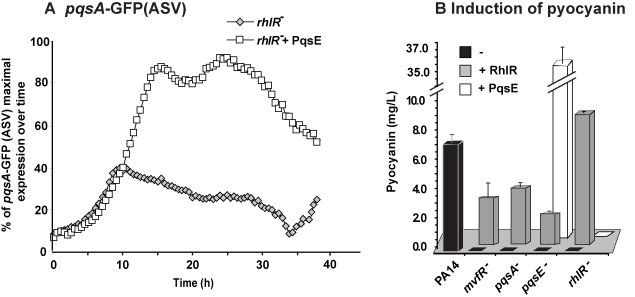
MvfR network regulation requires finely tuned cooperation between the MvfR component PqsE and the AHL QS regulator RhlR. (A) The expression of *pqsA* was determined by measuring GFP emission. A *pqsA*-GFP (ASV) fusion in the *rhlR* mutant harboring pDN19*pqsE* was used to determine *pqsA* expression levels. (B) Pyocyanin levels were measured from various PA14 mutants harboring either pDN19*pqsE* or pUCP20*rhlR* plasmids. Empty vector served as control.

To determine whether there was indeed a functional relationship between the respective communication-systems components RhlR and PqsE in the regulation of the MvfR regulon signal production and whether they together affected signal integration, we proceeded to assess whether there was a RhlR-PqsE codependency in the negative regulation of HAQ biosynthesis. Figures 3A and S4B show that overexpression of PqsE in a *rhlR* ^−^ mutant did not result in a downregulation of the promoter-derived expression of the *pqs* operon in contrast to the overexpression of PqsE in the wild-type (WT) strain PA14 where expression of the *pqs* operon was downregulated (Figure 2 and Figure S2D). These results indicate that PqsE negative control of the activity of the MvfR regulon depends on RhlR.

Second, we examined whether there was an RhlR-PqsE codependency in signal integration by MvfR-regulon virulence genes downstream of PqsE. To this end, we assessed whether PqsE overproduction in *rhlR* ^−^ cells could restore pyocyanin production since it was completely abolished in both *pqsE^−^* [11],[19] and *rhlR^−^* [38] mutants. Figure 3B shows that PqsE did not restore pyocyanin production in *rhlR* ^−^ while RhlR expression partially (∼30%) restored pyocyanin production in *pqsE^−^* mutant cells. This finding suggests that PqsE also depends on RhlR in the positive regulation of pyocyanin production and that RhlR acts downstream of PqsE. Interestingly, Figure S5 shows that pyoverdine levels are higher in *rhlR* ^−^ than in PA14 but not in *pqsE^−^* mutant cells. Moreover, PqsE or RhlR overproduction in *rhlR* ^−^ or *pqsE^−^* mutant cells respectively did not fully downregulated pyoverdine production, while PqsE or RhlR overproduction in the corresponding mutant cells did (Figure S5). This finding suggests RhlR-PqsE codependency in the homeostatic regulation of pyoverdine.

Based on the above findings, it is likely that the PqsE-RhlR activities were not limited to controlling downstream genes associated only with pyocyanin or pyoverdine production if the high number of genes co-regulated by PqsE and the Las/Rhl system are considered (Figure S3A).

### Signal integration studies reveal a homeostatic negative feedback regulation by HHQ and PQS on cell-cell signaling and PqsE-controlled genes, respectively

**Figure 4:**
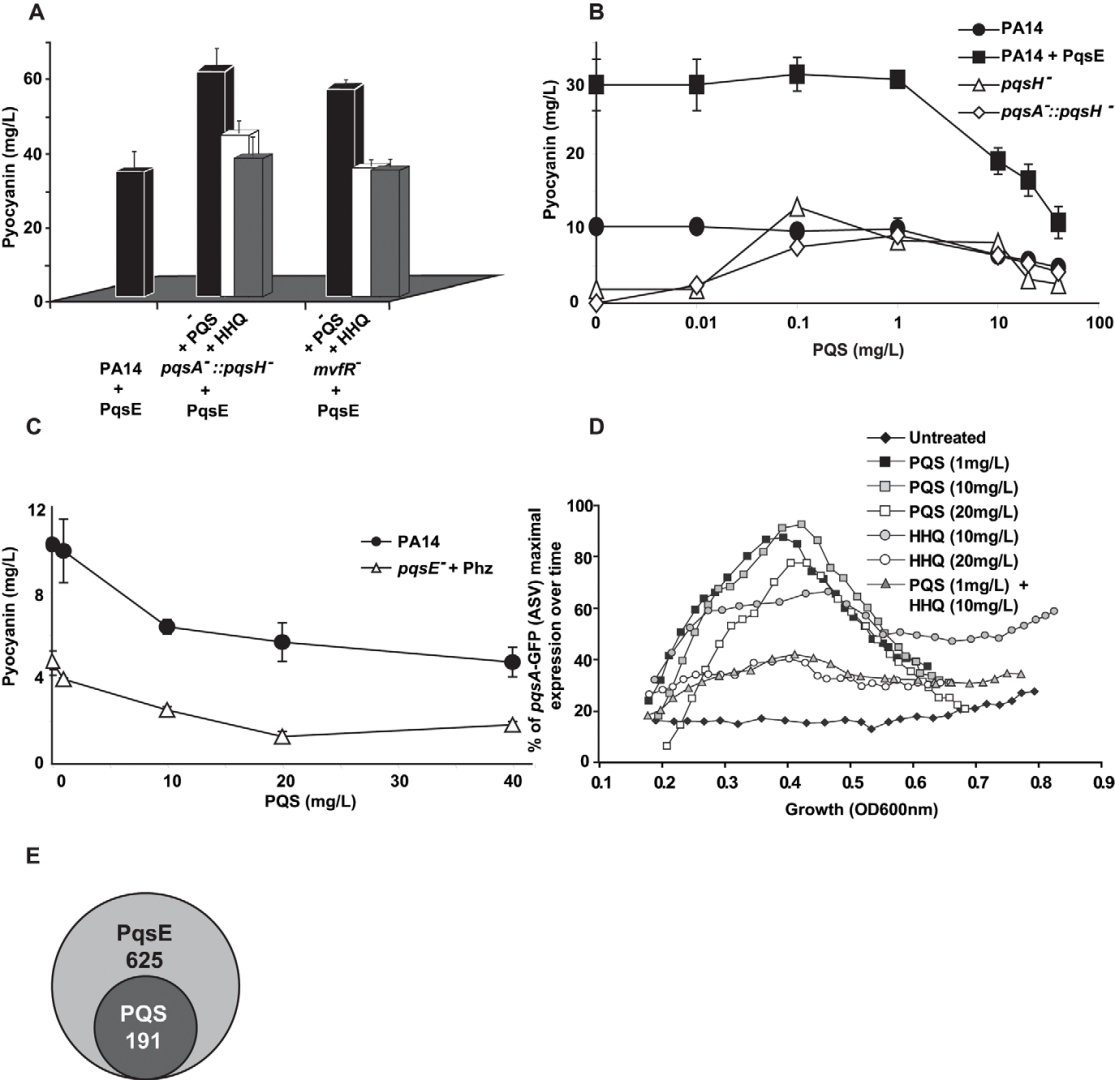
Negative homeostatic feedback regulation on MvfR regulon products and activity is mediated via cell-cell signaling molecule concentration. (A) Pyocyanin levels were assessed in PA14 and mutants cells harboring the plasmid pDN19*pqsE* with or without the addition of PQS or HHQ (20 mg/L). t-tests (p<0.05) showed that the difference between untreated and PQS/HHQ treated cells was statistically significant (B–C) Pyocyanin levels were determined following the addition of PQS over a broad-range of concentrations using a PQS non-producing strain (B) or using a narrow range of PQS concentrations in PQS-producing strains (C). PqsE was constitutively expressed (+PqsE). The empty vector was used as a control. *phz* genes were expressed following co-culture of *pqsE^−^* cells constitutively expressing *phzA2-G2* with *pqsE^−^* cells constitutively expressing the *phzM and phzS* genes. The cells were grown in the presence of exogenously added PQS and pyocyanin production measured by measuring the OD_600 nm_. (D) The expression of *pqsA* was determined using a *pqsA*-GFP (ASV) fusion in a *pqsA-::pqsH^−^* double mutant in the presence of various concentrations of HHQ and PQS. (E) A Venn diagram showing the number of PqsE-regulated genes counterbalanced by PQS. The data was adapted from Table S1.

The pyocyanin levels produced by the non-HAQs producing mutants *pqsA* ^−^, *mvfR* ^−^ and AA^−^ [12],[19],[22],[53] overexpressing *pqsE* were higher than the levels produced by the HAQs-producing PA14 parental strain carrying the same plasmid (Figure 1A). This difference raised the question regarding whether the presence and/or levels of HAQs had dose-dependent negative effects on pyocyanin levels. To this end we assessed the effect of exogenously-added HAQs on pyocyanin levels by using 20 mg/L of PQS or HHQ, a concentration corresponding to the approximate maximal physiologic levels reached by PA14 or *pqsH* ^−^ strains respectively at stationary phase ([17] and Figure S4A). Figure 4A shows that the pyocyanin levels in either *pqsA::pqsH* ^−^ or *mvfR* ^−^ mutants overexpressing *pqsE* were significantly lower in the presence of either HHQ or PQS. Figure 4B shows that PQS concentrations (up to 1 mg/L) induced pyocyanin production in both *pqsH* ^−^ and *pqsA^−^::pqsH* ^−^ cells but concentrations >1 mg/L decreased pyocyanin production in a dose-dependent manner in all strains tested (Figure 4B) without significantly affecting cell growth (data not shown). This concentration-dependent decrease in pyocyanin levels was independent of PqsE function and *phz* operon regulation since it was also observed in *pqsE^−^* cells constitutively expressing *phz* genes (Figure 4C). The PQS-mediated down-regulation was not specific to PA14 cells as it was also observed in the PA01 *P. aeruginosa* strain (Figure 4C).

To determine whether high physiological levels of PQS and/or HHQ negatively-impact *pqs* operon gene expression, we conducted experiments using *pqsA* ^−^::*pqsH* ^−^ cells harboring the *pqsA*-GFP (ASV) reporter gene. Figure 4D shows that 20 mg/L HHQ negatively-impacted *pqsA* gene expression compared to 10 mg/L. *PqsA* gene expression was not affected by any of the PQS concentrations tested. Interestingly, a negative effect on *pqsA* gene expression, similar to that observed following treatment with 20 mg/L HHQ, was also observed when the two HAQs were added together in sub-inhibitory concentrations (1 mg/L PQS +10 mg/L of HHQ). This result is indicating that together HHQ and PQS have synergistic inhibitory effect and implying also that high activation of the *pqs* operon led to its down-regulation.

To further elucidate the role of PQS on PqsE-dependent gene regulation, we compared the transcriptional profiles of *mvfR* ^−^ mutant cells overexpressing PqsE in the absence or presence of 20 mg/L PQS (Table S1). High PQS concentrations negatively affected the expression of 191 of 625 (31%) PqsE-regulated genes (Figure 4E and Table S1). This effect was more apparent among the known and putative virulence factors where the expression of 64% of the PqsE-regulated genes, (including chitinase, halovibrin, cellulase, pyocins, lectin, and elastase genes) was significantly reduced by more than 2-fold upon PQS addition (Table S1). The addition of PQS further increased the expression of only 7 genes: *fpvA*, the major pyoverdine receptor; *gatC*, a Glu-tRNA amidotransferase subunit C; *sucA*, a 2-oxoglutarate dehydrogenase; *bkdA1*, a 2-oxoisovalerate dehydrogenase and of three hypothetical proteins; PA4642, PA1343 and PA2405 (Table S1). Interestingly, transcription of *phz* operon genes was not modified by the addition of PQS although pyocyanin production was affected (Figure 4A), suggesting that PQS may be acting post-transcriptionally in this case.

### Homeostatic feedback modulation of the MvfR regulon is fine-tuned by an iron starvation response

As shown in Table S3A, PqsE positively-affected the expression of 43 iron starvation-related genes [36],[37] including the iron starvation sigma factor PvdS [41],[60], the pyochelin regulator PchR [61], *vqsR* [31],[62] and PA2384 [63]. Interestingly, PqsE negatively regulated only 6 iron related genes, *bfrB* and the siderophore pyoverdine associated genes *pvdA pvdF*, *pvdJ*, *pvdN and pvdQ* (Table S3A) reflected also in the pyoverdine levels (Figure S5). It is noteworthy that PqsE acted differentially on the siderophores, serving as a positive regulator of pyochelin and a negative regulator of pyoverdine (Figure S5). In addition, Table S3A reveal that HAQs are also involved in the control of iron-related genes by PqsE since constitutive expression of *pqsE* triggered this effect in the *mvfR* ^−^ background cells lacking HAQs but not in PA14 cells.

**Figure 5:**
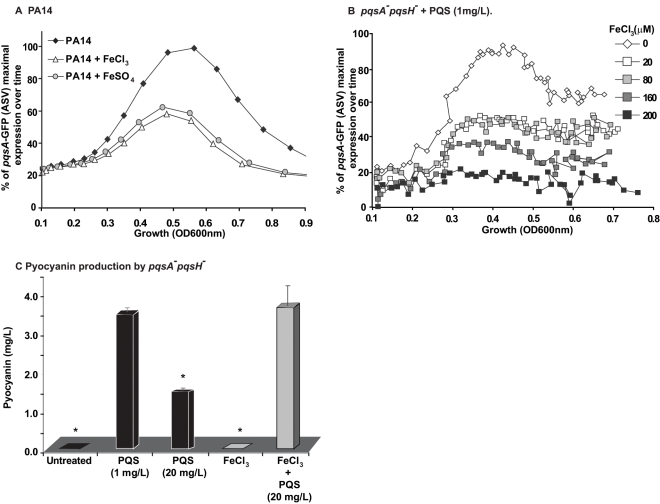
Homeostatic interplay between PQS and iron: Iron fine-tunes PQS activities. The effect of iron on MvfR induction was tested using the *pqsA*-GFP reporter in PA14 (A) and PA14 *pqsA* ^−^::*pqsH* cells treated with PQS (1 mg/L) (B). The effect of iron on pyocyanin production was tested when PQS was supplied at 1 mg/L or 20 mg/L (C). The cells were grown in low iron medium D-TSB or in media supplemented with iron (FeCl_3_ or FeSO_4_, 200 µM). Asterisks show samples that are statistically significant different (P value<0.05) from the PQS 1 mg/L treated sample.

To examine how iron starvation is translated in the context of MvfR signaling, we first examined whether there is a relationship between iron starvation and the regulation of PQS and MvfR regulon genes. We compared *pqsA* transcription using a *pqsA*-GFP (ASV) reporter in PA14 cells grown in the absence (D-TSB medium) or presence of high iron levels. Figure 5A demonstrates that iron significantly reduced *pqsA* transcription. Subsequently, we examined the effect of iron directly on the induction of *pqs* operon transcription in presence only of PQS and not of other HAQs in *pqsA* ^−^::*pqsH^−^* mutant cells. Using 1 mg/L PQS, an amount sufficient to fully induce *pqs* operon transcription and increasing concentrations of FeCl_3_ Figure 5B shows an iron concentration-dependent effect on *pqsA* gene expression.

We next examined if iron could also counterbalance the downstream effects of PQS on PqsE-dependent genes by assessing the effect of HAQs and iron on pyocyanin production. Figure 5C shows that the addition of iron abolished the reduction in pyocyanin production conferred by PQS (20 mg/L) and restored pyocyanin production to that observed in the presence of 1 mg/L PQS. A similar effect was observed in PA14 cells and *pqsA^−::^pqsH^−^* cells overexpressing PqsE (Figure S6A) where the addition of 20 mg/L PQS decreased pyocyanin levels which were restored in the presence of iron. Since iron alone did not affect pyocyanin production in the experimental conditions used, it suggested that pyocyanin production was affected due to direct effect of iron on PQS. No significant difference in growth was observed between PA14 cells grown in absence or presence of various concentrations of iron (up to 250 µM, Figure S6B). Collectively, these findings indicate that iron counterbalanced PQS-dependent regulation by ‘fine-tuning’ its activity, possibly by reducing PQS activity when it is in a complex with it.

## Discussion

In this work, we delineated paradigmatically the complex relationships between bacterial multi-layered regulatory QS circuitries, their signaling molecules, and the environmental cues to which they respond.

The intracellular communication system of *P. aeruginosa* possesses complex signal transduction systems allowing this versatile pathogen to regulate and coordinate virulence functions in the context of multiple hosts, environments, and competition from other microorganisms [7],[64],[65],[66]. Here we showed that one of these complex signal transduction systems, MvfR, responds to both positive and negative feedback loops that are interconnected with the RhlR QS complex system and that these interactions fine tune the production and concentration of secreted output signals that in turn serve as inputs to preserve a homeostatic regulation. Moreover, our experiments demonstrated that via the finely tuned cooperation and homeostatic interplay between the MvfR circuitry components PqsE, and PQS and HHQ with RhlR and iron, this pathogen governs and balances the intensity of its virulence response.

Although HHQ and PQS principally serve as MvfR ligands [17],[18], our results show that once maximal *in vitro* physiological levels are reached, they negatively impact their own production and the downstream PqsE regulated genes. PqsE, HHQ and PQS are essential molecules in the negative feedback auto-regulatory loops that contribute to this homeostatic regulation. Although the HHQ concentrations shown here are not attained *in vitro* because HHQ is fully converted into PQS, this effect is most likely relevant *in vivo* where we have shown that HHQ levels are higher than those of PQS [17]. In addition, in *lasR* ^−^ mutants that accumulated during chronic infections HHQ levels are also higher than PQS since PqsH responsible for the conversion of HHQ to PQS is under the control of LasR [67]. Nevertheless, we show that HHQ and PQS have together synergistic effect as a negative auto-regulators that down-regulated *pqs* operon transcription, reducing their own production and that of the other HAQs. Thus, jointly with PqsE, PQS and HHQ most probably contributed to the down-regulation of the *pqs* and *phn* operons observed during the late growth phase of *P. aeruginosa* (Figure S1).

In addition to being activator and auto-down-regulator PQS acted also as a homeostatic agent at high physiological concentrations by down-regulating most PqsE-dependent, downstream genes. Consistently, maximum pyocyanin production occurred only at low PQS concentrations that were sufficient to maximally activate the *pqs* operon. The homeostatic effect of PQS downstream of the PqsE genes was clearly independent of MvfR, PqsE and of other HAQs given that its effects were still apparent in *pqsA^−^* and *mvfR^−^* backgrounds. Interestingly, this effect appeared also to be post-transcriptional since PQS did not significantly impact *phz* operon transcription but affected pyocyanin production even when the *phz* operon was constitutively expressed. The mechanism behind this effect remains to be discovered. One intriguing possibility may be that PQS exerts its effect *via* RsmA and/or on small RNAs like *rsmZ* or *prrF*.

Previous studies have suggested that while PqsE is the PQS response protein [19],[20], it does not influence PQS production [11],[12]. Here we show that PqsE is a crucial player in orchestrating the homeostatic regulation of the signaling molecules HHQ and PQS as well as establishing a connection to the QS RhlR system, underscoring it as a key mediator of MvfR regulon activation and cooperation with the AHL QS system. Our findings also provide initial answers as to why PqsE, although not involved in the synthesis of HAQs *in vivo* or *in vitro* [11],[19],[20]), is tightly regulated together with the other *pqs* operon genes. Although our findings are primarily based on *trans*-regulatory studies, the overexpression of PqsE demonstrated for the first time that PqsE can impact HAQs concentrations by down-regulating their production. In corroboration, are both the AA accumulation and the transcriptional induction of the *antABC* genes responsible for AA degradation [68],[69] and shown to be regulated by *prrF1* and *prrF2* [45]. Since *pqsE* is co-transcribed by MvfR together with *pqsA-D* genes, the reduced production of HAQs mediated by PqsE indicates that *pqsE* gene transcription itself is also downregulated in a negative feedback mechanism that finely balances the regulatory loop.

Although PQS and HHQ signal molecules are critical to MvfR-dependent gene expression, their addition has failed to rescue *pqsE* ^-^ mutant cells to activate expression of many MvfR-regulated genes or to produce of pyocyanin [11],[17],[19],[20]. Here we found that overexpression of PqsE induced pyocyanin production and transcription of an additional approximately 600 MvfR-regulated genes independently of MvfR, HAQs and AA, demonstrating the crucial role of PqsE in activating MvfR regulon genes independently of the HAQs. Ultimately, expression of PqsE in an *mvfR* ^−^ or *pqsA* ^−^ strain restored *P. aeruginosa* virulence as determined by growth inhibition of yeast and flies feeding assay, indicating that PqsE did not need HAQs to confer virulence in these systems. Corroboratory results were reported by Farrow *et al.* [51] who showed in a qualitative manner that expression of PqsE in an *mvfR* ^−^ mutant restored pyocyanin production. These results together indicate that, at least with regard to the genes listed in Table S1, PQS and HHQ only act as inducers of MvfR to express PqsE that once expressed induces the *P. aeruginosa* virulence response without HAQs or MvfR. Thus, PqsE cannot be designated as the “quinolone signal response protein”. Nevertheless, it is not yet known how PqsE, a protein that belongs to the metallo-beta-lactamase super family without any known DNA binding motifs, regulates the transcription of so many genes. Its predicted hydrolase activity suggests that it may cleave or participate in the synthesis of small molecules. Due to the location of the *pqsE* gene in the *pqs* operon, the immediate candidates likely targeted by PqsE are HAQs. However following extensive LC/MS analyses, we were unable to detect any molecule that accumulated or diminished in concentration in *pqsE^−^* cultures compared to WT cultures (data not shown). In addition we were unable to complement pyocyanin production in a *pqsE^−^* culture by exogenously adding HAQs, AHLs or whole PA14 supernatants ([11] and data not shown). Nonetheless, collectively, our results indicate that PqsE is involved in a negative feedback loop that affects the regulation and integration of HAQs-mediated cell-cell signaling molecules and that is functionally dependent on RhlR. The exact nature of the co-dependency between PqsE and RhlR remains unclear. The downregulation of rhlR expression by ∼2 fold in a *pqsE* mutant is not sufficient to explain the striking transcriptional and phenotypic effects mediated by PqsE. Since PqsE is not predicted to be a transcriptional factor [50] it is highly likely that it may exert its effect on RhlR post-transcriptionally, and this effect may be perhaps extended to other transcriptional factors.

The MvfR affected gene list has a substantial overlap [11] with the previously published list of Rhl/Las-controlled genes [42], and the expression of almost all MvfR-regulated genes controlled by PqsE. Both PqsE activities (*i.e.*, fine-tuning HAQs production by down-regulating the *pqs* operon, induction of pyocyanin production and downregulation of pyoverdine production) were dependent on RhlR apparently acting downstream but in a tight collaboration with PqsE. Recently, Farrow and colleagues showed that the addition of AHL C_4_-HSL (a RhlR inducer) to PAO1 *pqsE* ^−^ isogenic mutants also restored pyocyanin production [51]. These findings, although we did not reproduce them in PA14 cells, are in agreement with our findings that PqsE and RhlR functions are linked. However, the exact relationship between PqsE and RhlR, that is when or how they cooperate, remains elusive since RhlR in some cases functions in the absence of PqsE; for example, the RhlR-dependent C_4_-HSL levels in a *pqsE* ^−^ mutant strain were identical to the parental strain (data not shown) as also was previously shown for the *mvfR^−^* mutant [11].

The relationship between iron, QS regulation, and *P. aeruginosa* virulence is multifaceted [31],[32],[34],[36],[45],[63] and extremely complex. Data presented in this report demonstrate that the MvfR regulon represents a striking paradigm of the interplay between environmental signals and bacterial secreted cell-cell signal molecules that participate in positive and negative homeostatic regulatory loops. QS MvfR components control the transcription of many iron related genes, while iron related regulators control the expression of QS genes (see Table S3B) in addition to iron related genes. The relationship between iron and QS regulation is further strengthened through the iron-related regulators VqsR [43] and the PA2384 product [63] that were found to control the expression of *phnAB* and *pqsA-E* operons. Furthermore, the iron starvation sigma factor PvdS was shown to positively control the expression of *mvfR* via its IS box [36], iron was shown to control the *pqs* operon during biofilm formation [32], and the two small Fur-regulated RNAs Prrf 1 and 2 positively-regulated PQS production [45]. Our results showing that iron levels affected HAQs activities both as inducers of MvfR and as fine-balancers provide corroboration for the view that the MvfR regulon is closely linked with iron regulation. The complexity of the interplay between the MvfR regulon and iron control is further increased by: a. the ability of PQS but not HHQ to trap iron [47], which likely reduces available iron within the cell and promotes iron starvation, thereby affecting PqsE-mediated control of bacterial iron response genes, including the siderophores pyochelin and pyoverdine; and b. iron, especially in high concentrations, induces oxidative stress that was shown to affect and being affected by PQS [70]. Thus, it is possible that some of the phenotypic effects of PQS and iron shown here could be attributed to oxidative stress. Thus, it would be of importance to further investigate the contribution of iron, as a nutrient, a signal molecule, and an oxidative stress inducer in QS and *P. aeruginosa* virulence.

The existence of a tight interconnection between iron concentrations, QS, and virulence in *P. aeruginosa* is likely due to iron conditions encountered *in vivo* [71],[72] serving as a signal indicating a hostile environment requiring expression of virulence or fitness-related genes. When host tissues become damaged as a consequence of virulence factor production, the resulting increase in iron concentrations should down-regulate virulence factor concentrations, thereby reducing bacterial virulence that may favor host survival and potentially chronic infection.

**Figure 6:**
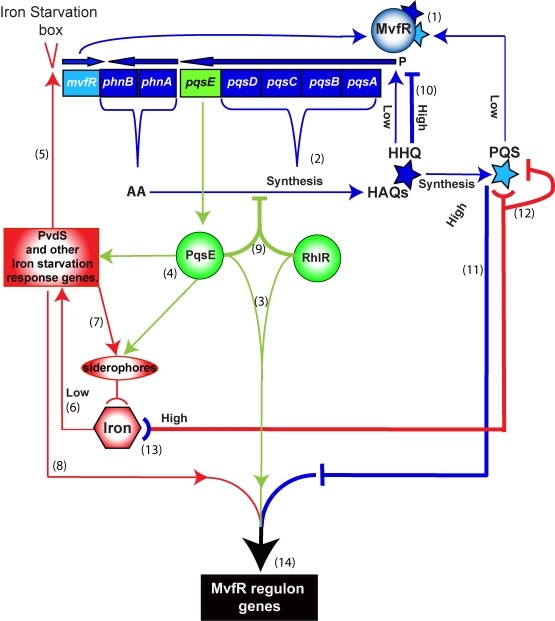
Schematic of the positive and negative homeostatic interplay among the MvfR regulon components PqsE, and PQS and HHQ with RhlR and iron. PqsE (green), HHQ and PQS (blue) and iron (red) play a dual role in up- or down-regulating the MvfR regulon. The outcome—that is the level of downstream gene expression translated into the bacterial virulence response—is the integrated sum of these interactions. Positive loops (thin lines): (1) MvfR is induced by HHQ and its derivative PQS to express *phn* and *pqs* operons, which are in turn (2) responsible for the synthesis of HAQs. PqsE is not required for HAQ synthesis and does not need AA or its derivatives for its “bottleneck” function, (3) controlling the expression of many virulence factors in cooperation with the AHL regulator RhlR. (4) PqsE also controls many iron starvation response genes, such as PvdS and siderophores. (5) PvdS in turn up-regulates the transcription of *mvfR* via an iron starvation box. (6) Low iron conditions also contribute to the induction of PvdS and other iron-related regulators to activate the iron response including (7) uptake of iron into the cell by siderophores as well as (8) induction of the virulence response. Negative loops (thick lines): (9) PqsE in cooperation with RhlR down-regulates the expression of the *phn* and *pqs* operons, thus reducing HAQ production. When a threshold concentration of HHQ is reached, (10) HHQ down-regulates the *pqs* operon. (11) PQS at high physiological levels in turn counterbalances the expression of PqsE-controlled genes, including many virulence factors. High levels of iron in presence of low levels of PQS, reduce *P. aeruginosa* virulence, at least in part, by (12) binding and inactivating PQS. In contrast, when PQS is at high physiological levels its inactivation by iron will increase virulence by reducing the negative PQS counterbalance and thus sustain the positive loops that include (13) iron starvation as a result of PQS trapping iron. (14) The integration of these processes enforces a fine-tuning of MvfR regulon gene expression levels, therefore determining the magnitude of virulence.

A complete understanding of the regulation of the multiple *P. aeruginosa* virulence networks, in particular the mechanisms of the homeostatic and down-regulation processes (Figure 6), will be essential for the development of drugs targeting QS inhibition [73],[74]. The findings presented in this study may aid in the design of anti-infective therapies tailored to interfere with virulence pathways and provide a paradigm for understanding the complex QS networks of other bacterial pathogens besides that of *P. aeruginosa*.

## Materials and Methods

### Bacterial strains, growth conditions, and plasmids

Table S4 lists bacterial strains and plasmids used in this study. *P. aeruginosa* were routinely grown in Luria Bertani (LB) broth at 37°C for 18 h, and diluted to OD_600 nm_ 0.05 and grown to the desired OD_600 nm_. For low iron media the bacteria were grown in D-TSB medium [36] that was treated with Chelex 100 beads (Bio-Rad, Hercules, CA) and for high iron FeCl_3_ or FeSO_4_ were added at concentrations of 200 µM. The *E. coli* JM109 strain was used for sub-cloning and plasmid propagation. The *E. coli* S17-1 strain was used for conjugation between *E. coli* and *P. aeruginosa* by the pEX18Ap-derivative allelic replacement method [75]. Antibiotics used included ampicillin (Amp) (100 µg/ml), carbenicillin (Crb) (300 µg/ml), gentamycin (Gnt) (15/60 µg/ml), kanamycin ((Kan), 50/200), tetracycline (Tet) (15/200 µg/ml) and chloramphenicol (Cam) (15/50 µg/ml) for *E. coli* and *P. aeruginosa* respectively.

### DNA manipulations

The plasmid overexpressing PqsE was generated by PCR amplification of the *pqsE* gene from PA14 genomic DNA using primer pairs GX119 and GX120 (Table S4). The PCR product was digested with *Hind*III/*Xba*I and sub-cloned into the pDN19 plasmid vector under *plac* promoter to generate pDN19*pqsE* that constitutively expresses *pqsE*. Construct integrity was confirmed by DNA sequencing. Plasmids were introduced into *E. coli* or *P. aeruginosa* PA14 by electroporation. Non polar deletions were generated by pEX18AP allelic replacement using sucrose selection. Fragments with the size of about 1 kb flanking the desired genes were cloned into the pEX18Ap plasmid vector and introduced into *E. coli* by electroporation followed by conjugation to *P. aeruginosa*. Alternatively, the λ-Red recombinase method was used to generate chromosomal deletions or insertions [53].

### Reporter genes

Two kinds of reporter genes were used: 1) translational and transcriptional fusions to *lacZ* where the β-galactosidase activity assay was performed in triplicate as described [76] and; 2) a *pqsA*-GFP (ASV) fusion consisting of a *pqsA* promoter upstream to a short-lived GFP that allows for the detection of *pqs* operon up or down regulation carried on the plasmid pAC37 [32]. Overnight cultures were diluted to an OD_600 nm_ of 0.05 in black, clear bottom sterile 96-well assay plates (Corning Inc., Corning, NY). The plates were incubated for 50 h at 37°C in an Infinite F200 plate reader (Tecan Group Ltd, Männedorf, Switzerland). Every 30 min the plates were shaken for 2 min and read at 600 nm and fluorescence detected by excitation at 485 nm and emission at 535 nm. The results are expressed as an average of 3–6 observations that were normalized to a strain that did not carry the plasmid pAC37.

### RNA isolation, generation and analysis of transcriptome data

Bacteria were respectively grown overnight at 37°C, diluted to an OD_600 nm_ of 0.05 in 25 ml LB with the corresponding antibiotics at 37°C until the OD_600 nm_ reached 3.0. The total RNA was isolated with the RNeasy Mini kit (QIAGEN Inc., Valencia, CA) and cDNA synthesis and labeling performed according to the manufacturer's instructions (Affymetrix, Santa Clara, CA). The *P. aeruginosa* PAO1 GeneChip® Genome array (Affymetrix) was used for hybridization, staining, washing and scanning according to the manufacturer's instructions. Experiments were independently performed in triplicate. Affymetrix DAT files were processed using the Affymetrix Gene Chip Operating System (GCOS) to create .*cel* files. The raw intensity .*cel* files were normalized by robust multi-chip analysis (RMA) (Bioconductor release 1.7) with PM-only models. Array quality control metrics generated by the Affymetrix Microarray Suite 5.0 were used to assess hybridization quality. Normalized expression values were analyzed with SAM (Significance Analysis of Microarray) using the permuted unpaired two-class test. Genes whose transcript levels exhibited either a 2-fold or up or down regulation and had a *q* value <6% were further analyzed. The results of the GeneChip® arrays were imported to GeneSpring 7.3 (Agilent Technologies, Inc., Palo Alto, CA) and the expression signals of the GeneChip® arrays were normalized to the constant value of 1.0 and the ratio cut-off was set to 2-fold. Annotations were performed using the database http://pseudomonas.com/. The transcriptome results were (in part) validated by assessing β-galactosidase expression and RT-PCR of selected genes (Figure S2). The data are deposited in NCBI GEO with accession number #GSE17147.

### Quantitative real-time RT- PCR

Cells from each triplicate experiment were harvested at an OD_600 nm_ of 2, 3 and 4. Total RNA was subsequently isolated using the RiboPure-Bacteria RNA Isolation kit (Ambion, Austin, TX) according to the manufacturer's instructions. cDNAs were synthesized with random reverse primers using the Reverse Transcription RETROscript kit (Ambion) according to the manufacturer's instructions. Specific primers (Table S4) for the amplification of products of approximately 200 base pairs were designed using the Primer3 algorithm (http://frodo.wi.mit.edu/primer3/) and analyzed by *In Silico* simulation of PCR amplifications (http://insilico.ehu.es/) and by the Primer Analysis Software NetPrimer (Premier Biosoft International, http://www.premierbiosoft.com/netprimer/index.html) for the detection of expressed *pqsA*, *pqsE* and *rpoD* that served as the normalizer genes [77]. Quantitative RT-PCR was carried out using the Brilliant II SYBR Green QPCR Master Mix (Stratagene) with a RT Fluorescence Detection System MX3005P (Stratagene, La Jolla, CA) in a 25 µl final volume. The efficiency of each pair of primers was determined by a standard curve of 8 dilutions of 1∶4 of PA14 genomic DNA. The relative expression ratios were calculated and analyzed using MXPro analysis software, version 4.01 (Stratagene) using a mathematical model that included an efficiency correction. The fold induction of mRNA was determined from the threshold values that were first normalized for *rpoD* gene expression that served as a normalizer and then for the threshold value of the WT strain harboring the pDN19 plasmid at an OD_600 nm_ of 2 that served as the calibrator. The data are expressed as the average of triplicate samples.

### HAQs detection

The quantification of HAQs concentration in bacterial culture supernatants and *in vivo* from rectus adominus muscle of burned and infected mice was performed by LC/MS as described previously [17],[78]. The HAQs were separated on a C18 reverse-phase column connected to a mass spectrometer using a water/acetonitrile gradient [78]. Positive electrospray in the MRM mode with 2×10^−3^ mTorr argon and 30 V as the collision gas were employed to quantify HAQs using the ion transitions HHQ 244>159, HHQ-D4 248>163, HQNO 260>159, PQS 260>175, and PQS-D4 264>179. The pseudomolecular ions of each compound were monitored in full scan mode using the unsaturated PA14 HAQs response factors.

### Pyocyanin production assay

Samples of 5 ml were spun down and the supernatants mixed with equal volumes of chloroform. The lower blue organic phase was collected and mixed with 5 ml of HCl (0.2 N). The upper reddish phase was collected and its OD_52 onm_ was measured. The concentration of pyocyanin was determined by the formula: mg/L  =  OD_52 onm_×17.072 normalized to cell counts and the statistical significance was assessed using the Student's 2 tailed *t*-test assuming equal variance [79]. In order to assess the production of pyocyanin by expression of the *phz* genes we used a co-culture of cells harboring the pUCP-A2G2 and pUCP-MS plasmids [80]. All experiments were performed in triplicate.

### Pyoverdine production detection

D-TSB medium was used to grow 200 µl of bacterial cells in 96 wells plate. Production of pyoverdine was assessed using a plate reader (Infinite F200, Tecan Group Ltd, Männedorf, Switzerland). Pyoverdine levels were determined every 30 minutes using excitation at 400 nm and emission at 460 nm and the values obtained were normalized to cell growth (OD_600 nm_). Pyoverdine concentrations were calculated using a calibration curve of fluorescence of a range of concentrations of pyoverdine (Sigma Aldrich, US).

### Yeast killing assay

Yeast (*Cryptococcus neoformans* KN99 α, *Candida albicans* ATCC #90028 DAY185 strain or *Saccharomyces cerevisiae* YJM310 strain) were plated for 2 days on YPD agar (Difco) plates at 30°C. A colony was picked and grown for 18 h in liquid YPD media (Difco) at 30°C with shaking (200 rpm). The yeast was diluted 1∶100 in 4 ml soft YPD agar (0.6% agar) and poured onto an YPD plate that was dried for 30 min in a laminar flow hood. A 1 µl drop of an overnight culture of the desired bacterial strain was put on top of the yeast lawn and the plate incubated for 2–3 days at 30°C. A dead yeast zone was formed around a by PA14 bacterial colony bun not around mutants such *e.g.*, *mvfR ^−^*, *pqsA ^−^* and *pqsE ^−^*. The viability of yeast in these zones was tested by plating yeast from distance of 5 mm from the bacterial colonies on YPD plates.

### Fly infection

Fly infection feeding assay was performed as previously described in [58],[59]. Briefly, 45 female Oregon-R flies per group, 5–7 days old, were fed with a mixture of 4 ml of LB bacterial culture at OD_600 nm_ 3.0 with 1 ml of 20% sucrose. Thus, feeding mix contained a final concentration of 80% LB containing ∼2×10^9^ bacterial cells per ml and 4% sucrose. An autoclaved cotton ball was placed at the bottom of each fly vial and was impregnated with 5 ml of the feeding mix. The 45 flies per treatment group were sub-divided in three fly vials (15 flies in each), sealed with a clean cotton ball, and incubated at 25°C. Fly survival was recorded twice a day until all flies succumbed to the infection. Statistical analysis of the survival curves was preformed using the log-rank test (Mantel-Haenszel) of the Kaplan-Meier estimate of survival using the software MedCalc (http://www.medcalc.be/). Two independent experiments gave similar results.

## Supporting Information

Figure S1 Transcription profile of *mvfR* and *pqsA-E*. The transcription profile was determined from the transcriptome analysis of PA14 cultures along the growth curve in LB at 37°C.
(0.85 MB EPS)

Figure S2 Microarray data validation. The effect of PqsE on the expression of various differentially-expressed genes in the transcriptome (Table S1) was further confirmed by β-galactosidase assays derived from transcriptional fusions of the tested genes with *lacZ* (A–C) and by quantitative PCR (D). The levels of *pqsA* and *pqsE* gene expression by PCR were determined from PA14 cultures harboring pDN19*pqsE* (+PqsE) or the control vector pDN19. The PA14 sample at OD_600 nm_ of 2 served as the calibrator.
(1.12 MB EPS)

Figure S3 PqsE and RhlR cooperate in the regulation of the *pqs* operon and of PqsE downstream genes. (A) A Venn diagram showing the number of genes co-regulated by PqsE (Table S1) and by the Las/Rhl system [42]. (B) Constitutively-expressed PqsE does not reduce the expression of *pqsA* in a *rhlR* ^−^ mutant. The expression of the *pqsA* gene in an *rhlR* ^−^ mutant constitutively expressing PqsE or harboring the empty vector was assessed by quantitative PCR reaction. An OD_600 nm_ reading of a sample from *rhlR* ^−^ served as the calibrator.
(0.82 MB EPS)

Figure S4 *pqsE* is not required for HAQs production. The levels of HHQ, PQS and HQNO were assessed by LC/MS from PA14 (circles) and *pqsE* ^−^ mutant (squares) cultures at various growth stages in LB at 37°C.
(0.68 MB EPS)

Figure S5 PqsE downregulates pyoverdine production in a RhlR dependent manner. The effect of PqsE and RhlR on pyoverdine production was assessed by measuring the pyoverdine production in PA14 and mutants harboring pDN19*pqsE* (+PqsE) or the empty vector pDN19 cells as control. Cells were grown in D-TSB medium in 96 wells plate and were incubated at 37°C with shaking for 1 minute every 30 minutes. The results shown are averages of 6 wells.
(4.36 MB EPS)

Figure S6 Iron counteracts PQS-mediated activity. (A) The effect of iron and PQS was assessed by measuring pyocyanin production in PA14 and a PA14 *pqsA::* ^−^ *pqsH* ^−^ double-mutant constitutively expressing PqsE. PQS was added at 20 mg/L and iron at 200 µM. Asterisks show samples that are statistical significantly different (P value<0.01) from the untreated sample of PA14 (*) and *pqsA::* ^−^ *pqsH* ^−^ (**). (B) The effect of iron is not a consequence of growth impairment. Growth curves were performed with PA14 cells in D-TSB media supplied with various concentrations of FeCl_3_ in 96 wells plate incubated at 37°C with shaking for 1 minute every 30 minutes. The results shown are averages of 6 wells.
(0.93 MB EPS)

Table S1 The PqsE controlled genes list. A list of genes comprising the PqsE regulated genes was generated from our transcriptional data (NCBI GEO accession number #GSE17147). The values represent ratios of differential expression between the *pqsE* ^−^ mutant vs. PA14 (*pqsE* ^−^), *mvfR* ^−^ vs. PA14 (*mvfR* ^−^), *mvfR* ^−^ harboring pDN19*pqsE* vs. *mvfR* ^−^ with pDN19 (*mvfR* ^−^ + PqsE), *mvfR* ^−^ + pDN19*pqsE* treated with PQS (20 mg/L) vs. untreated (*mvfR* ^−^ + PqsE + PQS) and PA14 harboring pDN19*pqsE* vs. PA14 harboring the empty vector pDN19 (PA14 + PqsE). The expression results were validated using reporter genes and quantitative PCR (Figure S2).
(0.08 MB PDF)

Table S2 Transcriptional regulators controlled by the MvfR pathway. The data on the differential expression of transcription regulators was adapted from Table S1.
(0.07 MB XLS)

Table S3 The interplay between the *pqs* operon and iron. The *mvfR* regulon components controlling (A) or controlled by (B) iron related regulators. (A) The data was adapted from Table S1. The values represent fold changes in the *pqsE* ^−^ mutant vs. PA14 (*pqsE* ^−^), *mvfR* ^−^ vs. PA14 (*mvfR* ^−^), *mvfR* ^−^ harboring pDN19*pqsE* vs. *mvfR* ^−^ + pDN19 (*mvfR* ^−^ + PqsE), *mvfR* ^−^ + PqsE treated with PQS (20 mg/L) vs. untreated (*mvfR* ^−^ + PqsE + PQS) and PA14 harboring pDN19*pqsE* vs. PA14 with pDN19 (PA14 + PqsE). (B) Iron related regulators controlling the MvfR regulon component. Fold change in expression of *mvfR, pqsA-E* and *phnAB* were retrieved from previously published studies of iron-related conditions and regulators ^1^ [37], ^2^ [36], ^3^ [62], ^4^ [63].
(0.07 MB XLS)

Table S4 Strains, plasmids and primers. The *P. aeruginosa* strains, plasmids and primers that were used in this study.
(0.07 MB DOC)
