# Supplementary material for: Homeostatic Interplay between Bacterial Cell-Cell Signaling and Iron in Virulence
Source: PLoS Pathog. 2010 Mar 12;6(3):e1000810. doi: 10.1371/journal.ppat.1000810 (PMC2837411; doi:10.1371/journal.ppat.1000810)
Supplement: Table S1 — The PqsE controlled genes list. A list of genes comprising the PqsE regulated genes was generated from our transcriptional data (NCBI GEO accession number #GSE17147). The values represent ratios of differential expression between the pqsE − mutant vs. PA14 (pqsE −), mvfR − vs. PA14 (mvfR −), mvfR − harboring pDN19pqsE vs. mvfR − with pDN19 (mvfR − + PqsE), mvfR − + pDN19pqsE treated with PQS (20 mg/L) vs. untreated (mvfR − + PqsE + PQS) and PA14 harboring pDN19pqsE vs. PA14 harboring the empty vector pDN19 (PA14 + PqsE). The expression results were validated using reporter genes and quantitative PCR (Figure S2). (0.08 MB PDF) [file ppat.1000810.s007.pdf]

MvfR and PqsE Controlled genes.

PA14 ID	PAO1 ortholog	Factor Name or Category	Gene name	*pqsE*$^-$	*mvfR*$^-$	*mvfR*$^-$ + PqsE	PA14 + PqsE	*mvfR*$^-$ + PqsE + PQS	PA14 + PqsE vs. *mvfR*$^-$ + PqsE	Description
**Known or putative virulence factors**										
**Positively regulated genes**										
PA14_48600	PA1874	**Adhesin**		-2.73	-5.59	4.17	-21.65	-4.20	-2.76	surface adhesion protein, putative
PA14_18520	PA3543	**Alginate**	*algK*			17.83		-10.91	-8.75	alginate biosynthetic protein AlgK precursor
PA14_69390	PA5255	**Alginate**	*algQ, algR2*	-2.22	-2.85				2.16	Alginate regulatory protein AlgQ
PA14_08230	PA2274	**Antibiotics**		-39.36	-15.28	21.68				hypothetical protein, Putative monooxygenase
PA14_05590	PA1876	**bacteriocin/lantibiotic**		-3.07	-4.39		-16.23			SunT, ABC-type bacteriocin/lantibiotic exporters, contain an N-terminal double-glycine peptidase domain [Defense mechanisms].
PA14_53290	PA2172	**Cellulase**		-5.38	-18.44	22.60	-4.98	-6.80	-3.43	FrvX, Cellulase M and related proteins [Carbohydrate transport and metabolism].
PA14_19350	PA3461	**Cellulase**	*yhfE*		-2.27	4.13	-7.25	-4.39	-3.31	FrvX, Cellulase M and related proteins [Carbohydrate transport and metabolism].
PA14_61040	PA2300	**Chitinase**	*chiC*	-8.61	-3.95	43.78		-6.67		Chitinase, 75% similar to chitinase of [Serratia marcescens]
PA14_18800	PA0852	**Chitin-binding protein**	*cbpD*	-4.31	-4.29	13.75		-3.58		chitin-binding protein CbpD precursor
PA14_48590	PA1871	**Elastase**	*lasA*	-3.53	-10.25	3.99	-6.71			LasA protease precursor
PA14_08200	PA3724	**Elastase**	*lasB*	-2.02	-2.10	3.22	-7.09	-3.22		LasB, Zinc metalloprotease (elastase) [Amino acid transport and metabolism]
PA14_47400	PA3909	**Extracellular nuclease**		-2.61	-2.23	3.31				COG2374, Predicted extracellular nuclease [General function prediction only]
PA14_38270	PA1914	**Halovibrin**	*hvn*	-10.43	-3.97	26.47	-62.50	-4.88	-2.16	conserved hypothetical protein, halovibrin, ADP-ribosyltransferases, 58% similar to hvn gene product of
PA14_66840	PA0122	**Hemolysin**		-3.13	-3.56	3.79			2.90	conserved hypothetical protein, 58% similar to Asp hemolysin [Aspergillus fumigatus].
PA14_48560	PA2367	**Hemolysin-coregulated protein**		-2.97	-2.33		-6.67		3.08	Hcp, Hemolysin-coregulated protein (uncharacterized) [Function unknown]
PA14_09220	PA2193	**Hydrogen cyanide**	*hcnA*	-5.82	-4.44	-4.52				hydrogen cyanide synthase HcnA
PA14_01490	PA2194	**Hydrogen cyanide**	*hcnB*	-4.33	-3.48	3.93		-3.07		hydrogen cyanide synthase HcnB
PA14_09300	PA2195	**Hydrogen cyanide**	*hcnC*	-5.34	-4.24					hydrogen cyanide synthase HcnC
PA14_55940	PA5481	**Inhibitor of vertebrate lysozyme**		-2.91	-2.10	3.39				hypothetical protein
PA14_36810	PA2570	**Lectin**	*lecA, pa1L*	-3.10	-9.99	7.29		-3.40		PA-I galactophilic lectin
PA14_20610	PA3361	**Lectin**	*lecB*	-5.02	-8.41					hypothetical protein
PA14_48540	PA1218	**Mitomycin antibiotics/polyketide fumonisin like**		-3.80	-2.44		-11.42			COG5285, Protein involved in biosynthesis of mitomycin antibiotics/polyketide fumonisin [Secondary metabolites biosynthesis, transport, and catabolism].
PA14_24180	PA0424	**Multi-drug resistance**	*mexR*	3.31	4.93		8.20			multidrug resistance operon repressor MexR
PA14_36330	PA1221	**Peptide, actinomycin like**		-4.41	-3.08		-6.62			EntF, Non-ribosomal peptide synthetase modules and related proteins, 49% similar to actinomycin synthetase II [Streptomyces chrysomallus], [Secondary metabolites biosynthesis, transport, and catabolism].

PA14_03490	PA1215	**Peptide, Polyketide**		-7.04	-2.00	4.04	-71.94			CaiC, Acyl-CoA synthetases (AMP-forming)/AMP-acid ligases II. 40% similar to a region of polyketide synthase [Amycolatopsis mediterranei] [Lipid metabolism / Secondary metabolites biosynthesis, transport, and catabolism].
PA14_14780	PA4078	**Peptide, saframycin Mx1 like**		-2.17	-3.97	13.05	-3.08	-5.81	-2.49	probable nonribosomal peptide synthetase, 47% similar to saframycin Mx1 synthetase A [Myxococcus xanthus].
PA14_00640	PA0051	**Phenazines**	*phzH*	-2.77	-2.29	9.29				AsnB, Asparagine synthase (glutamine-hydrolyzing) [Amino acid transport and metabolism].
PA14_03520	PA4221	**Pyochelin**	*fptA*	-161.20	-2.99	4.79	-5.99	-3.56	-3.48	Fe(III)-pyochelin receptor precursor
PA14_34870	PA4222	**Pyochelin**	*pchI*	-111.70	-4.50		-9.43			MdlB, ABC-type multidrug transport system, ATPase and permease components [Defense mechanisms].
PA14_21530	PA4223	**Pyochelin**	*pchH*	-40.02	-3.29		-6.13			MdlB, ABC-type multidrug transport system, ATPase and permease components [Defense mechanisms].
PA14_35160	PA4224	**Pyochelin**	*pchG*	-1740.00	-2.82	-2.48	-13.70			PchG, Oxidoreductase (NAD-binding), involved in siderophore biosynthesis [Secondary metabolites biosynthesis, transport, and catabolism].
PA14_58040	PA4225	**Pyochelin**	*pchF*	-277.10	-5.08		-12.44			pyochelin synthetase
PA14_61200	PA4226	**Pyochelin**	*pchE*	-122.10	-6.89	3.71	-14.10		-3.30	EntF, Non-ribosomal peptide synthetase modules and related proteins [Secondary metabolites biosynthesis
PA14_60500	PA4227	**Pyochelin**	*pchR*	-2.73	-8.65	2.74	-4.02		-2.00	transcriptional regulator PchR
PA14_68440	PA4228	**Pyochelin**	*pchD*	-2.72	-4.74	5.17	-20.24		-4.19	pyochelin biosynthesis protein PchD
PA14_64460	PA4229	**Pyochelin**	*pchC*	-2.08	-4.24		-13.74		-2.29	pyochelin biosynthetic protein PchC
PA14_53250	PA4230	**Pyochelin**	*pchB*	-90.32	-4.02		-8.93		-2.20	salicylate biosynthesis protein PchB
PA14_55780	PA4231	**Pyochelin**	*pchA*	-80.47	-5.59	3.05	-9.01			MenF, Isochorismate synthase [Coenzyme metabolism / Secondary metabolites biosynthesis, transport, and catabolism].
PA14_36730	PA0633	**Pyocin F2**			-3.11	3.54	-5.24		-2.37	hypothetical protein
PA14_37745	PA0635	**Pyocin F2**		-5.29	-14.76	10.15	-5.26	-6.13	-3.37	hypothetical protein
PA14_08050	PA0636	**Pyocin F2**		-2.41	-2.28	4.68	-4.50	-6.54	-3.45	COG5281, Phage-related minor tail protein [Function unknown].
PA14_37760	PA0637	**Pyocin F2**			-3.42	3.83	-5.05	-3.17	-2.48	COG4718, Phage-related protein [Function unknown].
PA14_16310	PA0638	**Pyocin F2**		-2.48	-3.67	3.12	-6.49	-3.62	-2.68	gp18, Phage-related protein [Function unknown].
PA14_56990	PA0639	**Pyocin F2**			-2.15	4.91	-3.60	-6.85	-2.57	COG1310, Predicted metal-dependent protease of the PAD1/JAB1 superfamily [General function prediction only].
PA14_08070	PA0616	**Pyocin R2**		-2.18	-2.26	2.86	-3.58			gpV, Phage P2 baseplate assembly protein gpV [General function prediction only]
PA14_09480	PA0617	**Pyocin R2**			-8.08	3.38	-3.86			COG3628, Phage baseplate assembly protein W [General function prediction only]
PA14_47210	PA0619	**Pyocin R2**			-2.99	3.02	-4.95			gpI, Bacteriophage P2-related tail formation protein [General function prediction only].
PA14_31350	PA0620	**Pyocin R2**		-2.21	-2.75	4.24	-3.08	-3.52	-2.23	COG5301, Phage-related tail fibre protein [General function prediction only].
PA14_08060	PA0621	**Pyocin R2**			-2.53	4.12		-4.76	-2.85	conserved hypothetical protein
PA14_60190	PA0622	**Pyocin R2**			-6.33	2.12	-6.90	-5.21	-3.50	COG3497, Phage tail sheath protein FI [General function prediction only].
PA14_42950	PA0623	**Pyocin R2**			-2.04	2.77	-3.61	-3.39	-2.13	COG3498, Phage tail tube protein FII [General function prediction only].
PA14_09260	PA0625	**Pyocin R2**			-2.50	3.07		-4.48	-2.26	COG3941, Mu-like prophage protein [General function prediction only]
PA14_08270	PA0629	**Pyocin R2**			-2.11	5.30	-9.90	-3.60	-5.62	COG3179, Predicted chitinase [General function prediction only].
PA14_48550	PA0632	**Pyocin R2**			-2.38	2.92		-3.19		hypothetical protein

PA14_14770	PA1901	**Pyocyanin**	*phzC2*	-5.92	-14.11	5.45				phenazine biosynthesis, AroG, 3-deoxy-D-arabino-heptulosonate 7-phosphate (DAHP) synthase [Amino acid transport and metabolism].
PA14_39925	PA1902	**Pyocyanin**	*phzD2*	-7.64	-20.76	4.87	-4.88			phenazine biosynthesis, EntB, Isochorismate hydrolase [Secondary metabolites biosynthesis, transport, and catabolism].
PA14_39910	PA1903	**Pyocyanin**	*phzE2*	-6.32	-7.94	6.42	-3.60			phenazine biosynthesis protein PhzE
PA14_39890	PA1904	**Pyocyanin**	*phzF2*	-5.81	-12.05		-4.42			phenazine biosynthesis, COG0384, Predicted epimerase, PhzC/PhzF homolog [General function [illegible]
PA14_72360	PA1905	**Pyocyanin**	*phzG2*	-5.46	-11.73	3.55	-3.48			PdxH, Pyridoxamine-phosphate oxidase [Coenzyme metabolism]
PA14_36780	PA4209	**Pyocyanin**	*phzM*	-2.83	-2.70					
PA14_39870	PA4210	**Pyocyanin**	*phzA1*	-28.09	-5.00	3.30			2.21	probable phenazine biosynthesis protein
PA14_09470	PA4211	**Pyocyanin**	*phzB1*	-10.89	-10.12	5.95				
PA14_09400	PA4217	**Pyocyanin**	*phzS*	-3.17	-14.96				3.00	probable FAD-dependent monooxygenase
PA14_33680	PA2398	**Pyoverdine**	*fpvA*		-27.10		-3.19	4.07		ferripyoverdine receptor, fpvA (ferripyoverdine receptor)
PA14_14710	PA3478	**Rahmnolipids**	*rhlB*	-4.07	-2.24	6.80	-5.65	-4.20	-2.20	rhamnosyltransferase chain B
PA14_09350	PA3479	**Rahmnolipids**	*rhlA*	-3.60	-3.14	5.77	-3.12	-3.22		rhamnosyltransferase chain A
PA14_34110	PA3477	**RhlR**	*rhlR*	-2.10	-2.54					transcriptional regulator RhlR
PA14_48060	PA1249	**Secreted alkaline protease**	*aprA*		-8.93		-10.85			alkaline metalloproteinase precursor
PA14_39945	PA2939	**Secreted aminopeptidases**	*pepB*	-2.45	-2.12	6.39	-14.60			Iap, Predicted aminopeptidases [General function prediction only].
PA14_01600	PA4175	**Secreted endoproteinase**	*piv, prpL*	-3.46	-4.98		-9.71			Endoproteinase Arg-C precursor; Pvds-regulated endoprotease, lysyl class
PA14_05510	PA0423	**Secreted protease**	*pasP, yceI*	-2.08	-7.42				3.31	conserved hypothetical protein
PA14_10340	PA4143	**Toxin transporter**	*cyaB, cvaB*	-2.02	-3.21					SunT, ABC-type bacteriocin/lantibiotic exporters, contain an N-terminal double-glycine peptidase domain [Defense mechanisms].
PA14_60400	PA1665	**Type VI secretion system**		-2.57	-11.38	2.00				COG3456, Uncharacterized conserved protein, contains FHA domain [Signal transduction mechanisms].
**Negatively regulated genes**										
PA14_51360	PA1001	**Anthranilic acid**	*phnA*	2.67	-1075.00		-8.85		4.69	TrpE, Anthranilate/para-aminobenzoate synthases component I [Amino acid transport and metabolism / Coenzyme metabolism].
PA14_51350	PA1002	**Anthranilic acid**	*phnB*	2.41	-20.19		-8.55			PabA, Anthranilate/para-aminobenzoate synthases component II [Amino acid transport and metabolism / [illegible]
PA14_51430	PA0996	**HAQs**	*pqsA*	2.48	-21.05		-4.59		3.15	Acs, Acyl-coenzyme A synthetases/AMP-(fatty) acid ligases [Lipid metabolism]
PA14_51420	PA0997	**HAQs**	*pqsB*		-49.64		-13.28		11.05	FabH, 3-oxoacyl-[acyl-carrier-protein].
PA14_51410	PA0998	**HAQs**	*pqsC*		-262.60		-9.35		10.01	FabH, 3-oxoacyl-[acyl-carrier-protein].
PA14_51390	PA0999	**HAQs**	*pqsD, fabH1*	2.11	-137.20		-10.93		3.57	FabH, 3-oxoacyl-[acyl-carrier-protein].
pqsE	***pqsE***									
**PA14_51380**	**PA1000**	**PqsE**	***pqsE***	**-12.58**	**-236.00**	**64.49**			**2.17**	**GloB, Zn-dependent hydrolases, including glyoxylases [General function prediction only].**

Other genes

Positively regulated genes

PA14_03550	PA0273	**Membrane proteins**				3.36		-3.95	-4.06	CynX, Cyanate permease, MFS transporter [Inorganic ion transport and metabolism].
PA14_03670	PA0281	**Membrane proteins**	*cysW*	-7.09	-5.05		3.06			CysW, ABC-type sulfate transport system, permease component [Inorganic ion transport and metabolism].
PA14_29710	PA1048	**Membrane proteins**		-2.59	-2.74		-6.06			OmpA, Outer membrane protein and related peptidoglycan-associated (lipo)proteins [Cell envelope biogenesis, outer membrane].
PA14_48570	PA1212	**Membrane proteins**		-2.24	-35.46	3.84	-6.06			MelB, Na+/melibiose symporter and related transporters [Carbohydrate transport and metabolism].
PA14_46240	PA1408	**Membrane proteins**			-2.67	2.05				COG3264, Small-conductance mechanosensitive channel [Cell envelope biogenesis, outer membrane].
PA14_42900	PA1669	**Membrane proteins**		-2.52	-2.46					IcmF, Uncharacterized protein conserved in bacteria [Function unknown].
PA14_40620	PA1848	**Membrane proteins**				9.14		-3.36	-3.57	AraJ, Arabinose efflux permease, MFS transporter [Carbohydrate transport and metabolism].
PA14_66880	PA2004	**Membrane proteins**		-2.13	-3.62		-6.25			GntT, H+/gluconate symporter and related permeases [Carbohydrate transport and metabolism / Amino acid transport and metabolism].
PA14_33570	PA2068	**Membrane proteins**		-5.71	-8.00	3.53	-3.03			hypothetical protein
PA14_05600	PA2148	**Membrane proteins**		-3.26	-2.27				-2.21	SapB, Uncharacterized membrane protein [Function unknown].
PA14_36080	PA2214	**Membrane proteins**				9.35		-12.15	-8.41	UhpC, Sugar phosphate permease [Carbohydrate transport and metabolism].
PA14_35020	PA2286	**Membrane proteins**			-2.30	5.07	3.06	-3.91	-2.48	HtpX, Zn-dependent protease with chaperone function [Posttranslational modification, protein turnover, chaperones].
PA14_40310	PA2327	**Membrane proteins**		-3.19	-3.24					TauC, ABC-type nitrate/sulfonate/bicarbonate transport system, permease component [Inorganic ion transport and metabolism].
PA14_13130	PA2331	**Membrane proteins**		-2.01	-3.13	-2.26	-3.41			COG2128, Uncharacterized conserved protein [Function unknown].
PA14_32720	PA2467	**Membrane proteins**	*foxR*	-2.75	-5.35	11.59			-2.62	FecR, Fe2+-dicitrate sensor, membrane component [Inorganic ion transport and metabolism / Signal transduction mechanisms].
PA14_34510	PA2868	**Membrane proteins**		-3.28	-6.85	-2.35				XtmA, Phage terminase, small subunit [DNA replication, recombination, and repair].
PA14_26360	PA2914	**Membrane proteins**				4.44		-5.10	-3.44	FepD, ABC-type Fe3+-siderophore transport system, permease component [Inorganic ion transport and metabolism].
PA14_26300	PA2919	**Membrane proteins**			-2.56	5.21		-5.59	-4.59	hypothetical protein
PA14_24760	PA3041	**Membrane proteins**	*yqjE*		-14.19	2.09	-7.19			COG5393, Predicted membrane protein [Function unknown].

PA14_21190	PA3311	**Membrane proteins**		-2.10	-7.79	3.68				COG5001, Predicted signal transduction protein containing a membrane domain, an EAL and a GGDEF domain [Signal transduction mechanisms].
PA14_20860	PA3340	**Membrane proteins**		-2.15	-2.07					FimV, Tfp pilus assembly protein FimV [Cell motility and secretion / Intracellular trafficking and secretion].
PA14_30620	PA3370	**Membrane proteins**		-2.34	-2.14	4.78		-5.88	-2.99	hypothetical protein
PA14_08160	PA3690	**Membrane proteins**		-2.12	-2.32	6.01	-3.64	-3.39	-2.17	ZntA, Cation transport ATPase [Inorganic ion transport and metabolism].
PA14_13040	PA3718	**Membrane proteins**		-11.98	-2.05	2.78				AraJ, Arabinose efflux permease [Carbohydrate transport and metabolism].
PA14_09520	PA4205	**Membrane proteins**	*mexG*	-87.50	-226.80	26.71				COG2259, Predicted membrane protein [Function unknown].
PA14_36570	PA4207	**Membrane proteins**	*mexI*	-33.35	-57.16	11.68				RND efflux transporter, AcrB, Cation/multidrug efflux pump [Defense mechanisms].
PA14_09470	PA4208	**Membrane proteins**	*opmD*	-18.74	-56.49	7.75	-5.32			TolC, Outer membrane protein [Cell envelope biogenesis, outer membrane / Intracellular trafficking and secretion].
PA14_39780	PA4218	**Membrane proteins**		-111.60	-11.46		-8.13			AraJ, Arabinose efflux permease [Carbohydrate transport and metabolism].
PA14_36740	PA4219	**Membrane proteins**	*yfpB*	-19.27	-18.84		-5.43		-2.29	PiuB, Uncharacterized iron-regulated membrane protein [Function unknown].
PA14_40230	PA4297	**Membrane proteins**	*tadG*	-2.05	-2.18	3.94				hypothetical protein
PA14_60960	PA4300	**Membrane proteins**	*tadC*	-2.16	-2.45		-3.33			hypothetical protein
PA14_40180	PA4614	**Membrane proteins**	*mscL*	-2.00	-2.07					MscL, Large-conductance mechanosensitive channel [Cell envelope biogenesis, outer membrane].
PA14_64240	PA4857	**Membrane proteins**		-2.22	-2.17					MarC, Multiple antibiotic transporter [Intracellular trafficking and secretion].
PA14_64680	PA4894	**Membrane proteins**			-6.24	89.57	-5.26	-12.80	-32.90	HupE, Hydrogenase/urease accessory protein [Posttranslational modification, protein turnover, chaperones].
PA14_64690	PA4895	**Membrane proteins**			-4.63	6.30		-5.08	-9.53	FecR, Fe2+-dicitrate sensor, membrane component [Inorganic ion transport and metabolism / Signal transduction mechanisms].
PA14_72180	PA5469	**Membrane proteins**			-6.52	11.75	41.72	-3.83	-6.16	TerC, Membrane protein TerC, possibly involved in tellurium resistance [Inorganic ion transport and metabolism].
PA14_16250	PA5482	**Membrane proteins**		-2.46	-6.82	3.32				hypothetical protein
PA14_00710	PA0059	**Adaptation, Protection**	*osmC*		-11.10	4.43				OsmC, Predicted redox protein, regulator of disulfide bond formation [Posttranslational modification, protein turnover, chaperones].
PA14_08040	PA0139	**Adaptation, Protection**	*ahpC*	-2.07	-2.23	3.26				AhpC, Peroxiredoxin [Posttranslational modification, protein turnover, chaperones].
PA14_24860	PA0140	**Adaptation, Protection**	*ahpF*	-2.89	-2.09	22.30		-6.76	-3.17	AhpF, Alkyl hydroperoxide reductase, large subunit [Posttranslational modification, protein turnover, chaperones].

PA14_02180	PA0173	**Adaptation, Protection**		-2.52	-8.96		-16.37			CheB, Chemotaxis response regulator containing a CheY-like receiver domain and a methylesterase domain [Cell motility and secretion / Signal transduction mechanisms].
PA14_02250	PA0178	**Adaptation, Protection**		-2.08	-9.49		-6.85			CheA, Chemotaxis protein histidine kinase and related kinases [Cell motility and secretion / Signal transduction mechanisms].
PA14_36520	PA0848	**Adaptation, Protection**		-12.59	-21.93	13.14		-9.43	-2.04	AhpC, Peroxiredoxin [Posttranslational modification, protein turnover, chaperones].
PA14_09240	PA2147	**Adaptation, Protection**	*katE*	-3.15	3.46	5.68	-8.70			KatE, Catalase [Inorganic ion transport and metabolism].
PA14_31810	PA2532	**Adaptation, Protection**	*tpx*	-2.61	-2.13	2.36				Tpx, Peroxiredoxin [Posttranslational modification, protein turnover, chaperones].
PA14_67350	PA2788	**Adaptation, Protection**		-2.11	-2.01					Tar, Methyl-accepting chemotaxis protein [Cell motility and secretion / Signal transduction mechanisms].
PA14_09150	PA4236	**Adaptation, Protection**	*katA, catA*	-2.15	-22.80	3.16	-3.51	-3.40		catalase
PA14_55900	PA4612	**Adaptation, Protection**	*ankB*	-5.45	-2.80	7.80		-5.43	-4.23	Arp, FOG: Ankyrin repeat [General function prediction only].
PA14_38260	PA4613	**Adaptation, Protection**	*katB*	-15.37	-7.41	11.73		-17.12	-4.97	catalase
PA14_23000	PA0430	**Amino acid biosynthesis**	*metF*	-4.30	-5.24					MetF, 5,10-methylenetetrahydrofolate reductase [Amino acid transport and metabolism].
PA14_34050	PA0432	**Amino acid biosynthesis**	*sahH*	-2.86	-3.61					SAM1, S-adenosylhomocysteine hydrolase [Coenzyme metabolism].
PA14_36770	PA0546	**Amino acid biosynthesis**	*metK*	-2.32	-7.87				2.36	methionine adenosyltransferase
PA14_08020	PA1217	**Amino acid biosynthesis**		-10.59	-2.24	3.54	-26.25			LeuA, Isopropylmalate/homocitrate/citramalate synthases [Amino acid transport and metabolism].
PA14_03090	PA1927	**Amino acid biosynthesis**	*metE*	-11.36	-12.85	9.96		-4.95	-3.20	5-methyltetrahydropteroyltriglutamate-homocysteine S-methyltransferase
PA14_19370	PA3459	**Amino acid biosynthesis**	*asnB*		-2.96	2.17				AsnB, Asparagine synthase (glutamine-hydrolyzing) [Amino acid transport and metabolism].
PA14_40290	PA3814	**Amino acid biosynthesis**	*iscS*	-10.67	-3.74	4.15				NifS, Cysteine sulfinate desulfinase/cysteine desulfurase and related enzymes [Amino acid transport and metabolism].
PA14_22990	PA5100	**Amino acid biosynthesis**	*hutU*	-2.26	-4.45		-5.35			urocanase
PA14_31370	PA5373	**Amino acid biosynthesis**	*betB*		-2.75	5.32				PutA, NAD-dependent aldehyde dehydrogenases [Energy production and conversion].
PA14_38825	PA1985	**Biosynthesis of cofactors**	*pqqA*		-4.13	4.19			-2.20	pyrroloquinoline quinone biosynthesis protein A
PA14_07090	PA1987	**Biosynthesis of cofactors**	*pqqC*	-2.15	-3.33					COG5424, Pyrroloquinoline quinone (Coenzyme PQQ) biosynthesis protein C [Coenzyme metabolism].
PA14_55820	PA3812	**Biosynthesis of cofactors**	*iscA*	-6.75	-4.24	4.09		-3.58	-2.41	probable iron-binding protein IscA
PA14_08240	PA3813	**Biosynthesis of cofactors**	*iscU*	-6.85	-3.97	5.15		-5.38	-3.70	IscU, NifU homolog involved in Fe-S cluster formation [Energy production and conversion].
PA14_64670	PA4893	**Biosynthesis of cofactors**	*ureG*		-2.75	7.72		-8.55	-7.38	HypB, Ni2+-binding GTPase involved in regulation of expression and maturation of urease and hydrogenase [Posttranslational modification, protein turnover, chaperones / Transcription].

PA14_65740	PA4973	**Biosynthesis of cofactors**	*thiC*			3.17		-4.29	-3.18	thiamin biosynthesis protein ThiC
PA14_02590	PA0212	**Carbon compound catabolism**	*mdcE*		-2.72	3.92	3.73	3.48	3.82	malonate decarboxylase gamma subunit
PA14_02760	PA0226	**Carbon compound catabolism**				4.77		-4.05	-3.15	AtoD, Acyl CoA:acetate/3-ketoacid CoA transferase, alpha subunit [Lipid metabolism].
PA14_37530	PA2086	**Carbon compound catabolism**		-2.99	-2.42					MhpC, Predicted hydrolases or acyltransferases (alpha/beta hydrolase superfamily) [General function prediction only].
PA14_36530	PA2290	**Carbon compound catabolism**	*gcd*	-2.88	-2.04		-6.71			Gcd, Glucose dehydrogenase [Carbohydrate transport and metabolism].
PA14_39925	PA2414	**Carbon compound catabolism**		-2.26	-2.53	5.24	-3.15	-3.69	-3.44	COG2133, Glucose/sorbosone dehydrogenases [Carbohydrate transport and metabolism].
PA14_33450	PA2416	**Carbon compound catabolism**	*treA*		-3.24	4.03			-2.42	periplasmic trehalase precursor
PA14_32240	PA2507	**Carbon compound catabolism**	*catA*		29.64			-33.33	-33.33	
PA14_32230	PA2508	**Carbon compound catabolism**	*catC*		3.43	11.90		-17.89	-17.37	CatC1, Muconolactone delta-isomerase [Secondary metabolites biosynthesis, transport, and catabolism].
PA14_32220	PA2509	**Carbon compound catabolism**	*catB*			43.79		-9.09	-16.67	
PA14_32160	PA2512	**Carbon compound catabolism**	*antA*		2.57	36.91		-48.31	-32.25	anthranilate dioxygenase large subunit
PA14_32150	PA2513	**Carbon compound catabolism**	*antB*		2.98	50.41		-56.18	-33.39	anthranilate dioxygenase small subunit
PA14_32140	PA2514	**Carbon compound catabolism**	*antC*		4.72	26.46		-86.96	-66.07	anthranilate dioxygenase reductase
PA14_49760	PA1130	**Cell wall / LPS / capsule**	*rhlC*		-3.04	6.05				COG1216, Predicted glycosyltransferases [General function prediction only].
PA14_46770	PA2717	**Central intermediary metabolism**	*cpo*	-2.79	-11.54		-7.94			MhpC, Predicted hydrolases or acyltransferases (alpha/beta hydrolase superfamily) [General function prediction only].
PA14_72370	PA5058	**Central intermediary metabolism**	*phaC*	-2.83	-2.39	3.41	-5.43			PhaC, Poly(3-hydroxyalkanoate) synthetase [Lipid metabolism].
PA14_68850	PA5213	**Central intermediary metabolism**	*gcvP1*	-2.06	-2.93		-4.59			GcvP, Glycine cleavage system protein P (pyridoxal-binding), C-terminal domain [Amino acid transport and metabolism].
PA14_26020	PA3810	**Chaperones and heat shock proteins**	*hscA*	-4.96	-106.30	9.24		-6.10	-5.49	DnaK, Molecular chaperone, heat shock protein [Posttranslational modification, protein turnover, chaperones].
PA14_36660	PA3811	**Chaperones and heat shock proteins**	*hscB*	-6.03	-3.60	5.04	-3.45		-3.08	DjlA, DnaJ-domain-containing proteins 1, heat shock protein [Posttranslational modification, protein turnover, chaperones].

PA14_57020	PA4386	**Chaperones and heat shock proteins**	*groES, mopB*	-2.94	-4.65	-2.56			2.68	GroS, Co-chaperonin GroES (HSP10) [Posttranslational modification, protein turnover, chaperones].
PA14_09930	PA4172	**DNA replication, recombination, modification and repair**		-2.21		3.07				XthA, Exonuclease III [DNA replication, recombination, and repair].
PA14_06830	PA0524	**Energy metabolism**	*norB*	-2.28	-12.64	7.57		-3.01		NorB, Nitric oxide reductase large subunit [Inorganic ion transport and metabolism].
PA14_61010	PA1317	**Energy metabolism**	*cyoA*	-4.32	-8.77	2.21	-3.89			CyoA, Heme/copper-type cytochrome/quinol oxidases, subunit 2 [Energy production and conversion].
PA14_09290	PA1318	**Energy metabolism**	*cyoB*	-4.99	-4.67		-4.27			CyoB, Heme/copper-type cytochrome/quinol oxidases, subunit 1 [Energy production and conversion].
PA14_09540	PA1319	**Energy metabolism**	*cyoC*	-8.27	-15.58		-16.37		-2.86	CyoC, Heme/copper-type cytochrome/quinol oxidase, subunit 3 [Energy production and conversion].
PA14_09530	PA1320	**Energy metabolism**	*cyoD*	-7.19	-10.91		-10.95			CyoD, Heme/copper-type cytochrome/quinol oxidase, subunit 4 [Energy production and conversion].
PA14_47150	PA1321	**Energy metabolism**	*cyoE*	-15.17	-2.87		-4.12			CyoE, Polyprenyltransferase (cytochrome oxidase assembly factor) [Posttranslational modification, protein turnover, chaperones].
PA14_55200	PA1562	**Energy metabolism**	*acnA, can*	-2.19	-8.86		-3.85	-4.59		AcnA, Aconitase A [Energy production and conversion].
PA14_36310	PA2153	**Energy metabolism**	*glgB*	-4.10	-6.27		-7.46	-3.77	-3.46	1,4-alpha-glucan branching enzyme
PA14_16660	PA2165	**Energy metabolism**	*glgA*	-2.23	-9.68	8.92			-2.13	GlgA, Glycogen synthase [Carbohydrate transport and metabolism].
PA14_09500	PA3032	**Energy metabolism**	*snr1*	-5.08	-11.98	10.83	-5.59	-5.59	-4.31	MauG, Cytochrome c peroxidase [Inorganic ion transport and metabolism].
PA14_23070	PA3183	**Energy metabolism**	*zwf*	-2.11	-4.17					Zwf, Glucose-6-phosphate 1-dehydrogenase [Carbohydrate transport and metabolism].
PA14_20180	PA3394	**Energy metabolism**	*nosF*		-12.35	11.73		-5.18	-4.66	CcmA, ABC-type multidrug transport system, ATPase component [Defense mechanisms].
PA14_48200	PA3415	**Energy metabolism**		-2.44	-2.02		-5.92			AceF, Pyruvate/2-oxoglutarate dehydrogenase complex, dihydrolipoamide acyltransferase (E2) component, and related enzymes [Energy production and conversion].
PA14_40630	PA3416	**Energy metabolism**		-3.03	-2.95	4.14	-26.95		-5.36	AcoB, Pyruvate/2-oxoglutarate dehydrogenase complex, dehydrogenase (E1) component, eukaryotic type, beta subunit [Energy production and conversion].
PA14_19900	PA3417	**Energy metabolism**			-2.29	4.33	-10.40		-2.34	AcoA, Pyruvate/2-oxoglutarate dehydrogenase complex, dehydrogenase (E1) component, eukaryotic type, alpha subunit [Energy production and conversion].
PA14_09340	PA3809	**Energy metabolism**	*fdx2*	-4.53	-2.42	5.21			-2.31	Fdx, Ferredoxin, [2Fe-2S] [Energy production and conversion].
PA14_62960	PA3929	**Energy metabolism**	*cioB*	-3.29	-3.23	2.02				AppB, Cytochrome bd-type quinol oxidase, subunit 2 [Energy production and conversion].
PA14_39890	PA3930	**Energy metabolism**	*cioA*	-4.43	-4.31		-6.41			CydA, Cytochrome bd-type quinol oxidase, subunit 1 [Energy production and conversion].
PA14_58030	PA4470	**Energy metabolism**	*fumC1*		-54.64	6.80			-4.84	fumarate hydratase

PA14_48610	PA1869	**Fatty acid and phospholipid metabolism**		-4.46	-2.55				2.67	AcpP, Acyl carrier protein [Lipid metabolism / Secondary metabolites biosynthesis, transport, and catabolism].
PA14_00650	PA0052	**Hypothetical, unclassified, unknown**			-2.64	3.30				COG1917, Uncharacterized conserved protein, contains double-stranded beta-helix domain [Function unknown].
PA14_00700	PA0058	**Hypothetical, unclassified, unknown**		-2.61	-2.11					COG3531, Predicted protein-disulfide isomerase [Posttranslational modification, protein turnover, chaperones].
PA14_19100	PA0250	**Hypothetical, unclassified, unknown**		-4.32	-3.76	7.35		-3.05		COG2905, Predicted signal-transduction protein containing cAMP-binding and CBS domains [Signal transduction mechanisms].
PA14_09270	PA0269	**Hypothetical, unclassified, unknown**		-3.05	-2.31	3.93		-4.08		COG2128, Uncharacterized conserved protein [Function unknown].
PA14_19910	PA0270	**Hypothetical, unclassified, unknown**		-2.66	-3.17	4.32	-3.03	-3.82	-2.16	COG1917, Uncharacterized conserved protein, contains double-stranded beta-helix domain [Function unknown].
PA14_22980	PA0271	**Hypothetical, unclassified, unknown**		-3.25	-4.32	4.30	-3.73	-3.14		COG1359, Uncharacterized conserved protein [Function unknown].
PA14_48530	PA0315	**Hypothetical, unclassified, unknown**		-3.09	-8.06		-4.42			hypothetical protein
PA14_52130	PA0429	**Hypothetical, unclassified, unknown**		-2.35	-2.65					hypothetical protein
PA14_09320	PA0431	**Hypothetical, unclassified, unknown**		-4.51	-4.01					SpoT, Guanosine polyphosphate pyrophosphohydrolases/synthetases [Signal transduction mechanisms / Transcription].
PA14_06120	PA0467	**Hypothetical, unclassified, unknown**		-2.23	-2.69		-4.85			Gst, Glutathione S-transferase [Posttranslational modification, protein turnover, chaperones].
PA14_61190	PA0468	**Hypothetical, unclassified, unknown**		-2.11	-4.47		-3.13			Vgb, Streptogramin lyase [Defense mechanisms].
PA14_07660	PA0587	**Hypothetical, unclassified, unknown**	*yeaH*		-2.17	4.95	-8.85	-7.09	-4.08	conserved hypothetical protein
PA14_07680	PA0588	**Hypothetical, unclassified, unknown**	*yeaG*		-21.01	4.42	-6.06	-3.97	-2.23	PrkA, Putative Ser protein kinase [Signal transduction mechanisms].
PA14_07980	PA0613	**Hypothetical, unclassified, unknown**		-4.11	2.57	6.45			2.47	hypothetical protein
PA14_55570	PA0673	**Hypothetical, unclassified, unknown**	*pigB*			9.13		-14.79	-10.34	hypothetical protein
PA14_54080	PA0788	**Hypothetical, unclassified, unknown**		-2.23	-2.97					MrcB, Membrane carboxypeptidase (penicillin-binding protein) [Cell envelope biogenesis, outer membrane].
PA14_53810	PA0808	**Hypothetical, unclassified, unknown**			-36.81	2.89				COG3832, Uncharacterized conserved protein [Function unknown].
PA14_52940	PA0875	**Hypothetical, unclassified, unknown**				4.92	3.29	-3.41	-2.33	COG1289, Predicted membrane protein [Function unknown].

PA14_72540	PA0938	**Hypothetical, unclassified, unknown**	-2.76	-3.59					WzzB, Chain length determinant protein [Cell envelope biogenesis, outer membrane].
PA14_59960	PA0982	**Hypothetical, unclassified, unknown**		-2.81	2.11				DsbG, Protein-disulfide isomerase [Posttranslational modification, protein turnover, chaperones].
PA14_49330	PA1166	**Hypothetical, unclassified, unknown**	-2.09	-5.78		-4.59			COG0412, Dienelactone hydrolase and related enzymes [Secondary metabolites biosynthesis, transport, and catabolism].
PA14_36500	PA1211	**Hypothetical, unclassified, unknown**	-9.80	-8.90		-11.39			
PA14_09370	PA1213	**Hypothetical, unclassified, unknown**	-5.07	-3.47		-8.26			hypothetical protein
PA14_36620	PA1214	**Hypothetical, unclassified, unknown**	-6.27	3.86		-11.03			AsnB, Asparagine synthase (glutamine-hydrolyzing) [Amino acid transport and metabolism].
PA14_09410	PA1216	**Hypothetical, unclassified, unknown**	-11.22	-2.16	3.84	-52.91			COG4976, Predicted methyltransferase (contains TPR repeat) [General function prediction only].
PA14_72510	PA1219	**Hypothetical, unclassified, unknown**	-3.34	-6.04	2.14	-3.68			COG3319, Thioesterase domains of type I polyketide synthases or non-ribosomal peptide synthetases [Secondary metabolites biosynthesis, transport, and catabolism].
PA14_09280	PA1220	**Hypothetical, unclassified, unknown**	-5.28	-3.12		-6.90		-2.14	hypothetical protein
PA14_38800	PA1289	**Hypothetical, unclassified, unknown**	-2.01	-3.24		-3.34			
PA14_47130	PA1323	**Hypothetical, unclassified, unknown**		-31.51	3.36	-8.13			ElaB, Uncharacterized conserved protein [Function unknown].
PA14_47120	PA1324	**Hypothetical, unclassified, unknown**		-11.26	2.32	-6.25			hypothetical protein
PA14_46840	PA1348	**Hypothetical, unclassified, unknown**		-3.21	4.46				hypothetical protein
PA14_46820	PA1350	**Hypothetical, unclassified, unknown**	-2.10	-3.07					COG4312, Uncharacterized protein conserved in bacteria [Function unknown].
PA14_11130	PA1353	**Hypothetical, unclassified, unknown**	-2.38	-2.36					PhnB, Uncharacterized protein conserved in bacteria [Function unknown].
PA14_34500	PA1354	**Hypothetical, unclassified, unknown**	-3.21	-3.75		-3.52			COG3795, Uncharacterized protein conserved in bacteria [Function unknown].
PA14_46750	PA1356	**Hypothetical, unclassified, unknown**	-2.05	-3.47	3.05				
PA14_43030	PA1658	**Hypothetical, unclassified, unknown**	-2.49	2.95					COG3517, Uncharacterized protein conserved in bacteria [Function unknown].

PA14_42920	PA1667	**Hypothetical, unclassified, unknown**			-10.06	2.42				COG3522, Uncharacterized protein conserved in bacteria [Function unknown].
PA14_42910	PA1668	**Hypothetical, unclassified, unknown**			-3.92	3.53				COG3455, Uncharacterized protein conserved in bacteria [Function unknown].
PA14_42130	PA1733	**Hypothetical, unclassified, unknown**		-2.10	-2.40		-3.33			COG3484, Predicted proteasome-type protease [Posttranslational modification, protein turnover, chaperones].
PA14_01580	PA1784	**Hypothetical, unclassified, unknown**		-2.63	-2.73		-3.56			
PA14_48630	PA1847	**Hypothetical, unclassified, unknown**	*yhgI*	-2.74	-2.01	3.96				COG0694, Thioredoxin-like proteins and domains [Posttranslational modification, protein turnover, chaperones].
PA14_40110	PA1887	**Hypothetical, unclassified, unknown**			-14.85	3.13	-5.00			hypothetical protein
PA14_55920	PA1888	**Hypothetical, unclassified, unknown**		-2.90	-2.39		-6.58			hypothetical protein
PA14_08090	PA1906	**Hypothetical, unclassified, unknown**		-2.04	-9.76	3.07				Hit, Diadenosine tetraphosphate (Ap4A) hydrolase and other HIT family hydrolases [Nucleotide transport and metabolism / Carbohydrate transport and metabolism / General function prediction only].
PA14_09210	PA1907	**Hypothetical, unclassified, unknown**		-2.17	-36.28	3.28		-5.03		COG4188, Predicted dienelactone hydrolase [General function prediction only].
PA14_39270	PA1951	**Hypothetical, unclassified, unknown**			-4.63	7.72				hypothetical protein
PA14_66850	PA2030	**Hypothetical, unclassified, unknown**		-3.59	-2.77	11.19		-6.25	-2.13	hypothetical protein
PA14_31360	PA2031	**Hypothetical, unclassified, unknown**		-4.85	-2.38	9.07		-6.90		hypothetical protein
PA14_38210	PA2034	**Hypothetical, unclassified, unknown**			-2.26	2.07			-4.25	CobL, Precorrin-6B methylase 2 [Coenzyme metabolism].
PA14_33900	PA2066	**Hypothetical, unclassified, unknown**		-2.92	-2.46					COG1434, Uncharacterized conserved protein [Function unknown].
PA14_36980	PA2134	**Hypothetical, unclassified, unknown**		-2.14	-3.92		-3.86			COG3652, Predicted outer membrane protein [Function unknown].
PA14_36880	PA2141	**Hypothetical, unclassified, unknown**			-2.54	7.70				CinA, Uncharacterized protein (competence- and mitomycin-induced) [General function prediction only].
PA14_36850	PA2143	**Hypothetical, unclassified, unknown**			-27.56	13.89				hypothetical protein
PA14_61520	PA2146	**Hypothetical, unclassified, unknown**	*yciG*	-2.33	-2.33	5.99		-5.52	-4.52	conserved hypothetical protein

PA14_04100	PA2149	**Hypothetical, unclassified, unknown**	-2.71	-2.03				-2.55	hypothetical protein
PA14_39590	PA2151	**Hypothetical, unclassified, unknown**	-2.55	-2.73	20.21	-5.15	-7.19	-8.89	AmyA, Glycosidases [Carbohydrate transport and metabolism].
PA14_14750	PA2159	**Hypothetical, unclassified, unknown**	-4.34	-5.26	4.25	-7.69		-2.10	COG0517, FOG: CBS domain [General function prediction only].
PA14_53300	PA2161	**Hypothetical, unclassified, unknown**	-2.66	-54.88	70.94	-6.71	-10.17	-7.38	hypothetical protein
PA14_08250	PA2163	**Hypothetical, unclassified, unknown**	-2.29	-2.58	3.95	-4.65			MalQ, 4-alpha-glucanotransferase [Carbohydrate transport and metabolism].
PA14_36300	PA2169	**Hypothetical, unclassified, unknown**	-3.52	-2.47		-4.50			hypothetical protein
PA14_39910	PA2171	**Hypothetical, unclassified, unknown**	-2.55	-4.07	9.61	-4.95	-8.40	-6.40	hypothetical protein
PA14_14800	PA2173	**Hypothetical, unclassified, unknown**	-2.87	-6.54	5.83	-6.06	-5.32	-3.62	hypothetical protein
PA14_36470	PA2174	**Hypothetical, unclassified, unknown**		-4.70	2.42				
PA14_36390	PA2179	**Hypothetical, unclassified, unknown**	-2.18	-2.49					HemK, Methylase of polypeptide chain release factors [Translation, ribosomal structure and biogenesis].
PA14_36375	PA2180	**Hypothetical, unclassified, unknown**	-2.18	-3.70	3.79		-3.08	-3.40	hypothetical protein
PA14_36350	PA2189	**Hypothetical, unclassified, unknown**	-2.70	-2.14					ClpP, Protease subunit of ATP-dependent Clp proteases [Posttranslational modification, protein turnover, chaperones / Intracellular trafficking and secretion].
PA14_47180	PA2328	**Hypothetical, unclassified, unknown**	-4.54	-5.47		-4.52			TauA, ABC-type nitrate/sulfonate/bicarbonate transport systems, periplasmic components [Inorganic ion transport and metabolism].
PA14_01720	PA2330	**Hypothetical, unclassified, unknown**	-11.90	-4.00		-6.80			CaiA, Acyl-CoA dehydrogenases [Lipid metabolism].
PA14_23030	PA2362	**Hypothetical, unclassified, unknown**	-2.24	-3.49		-6.80			COG3455, Uncharacterized protein conserved in bacteria [Function unknown].
PA14_34490	PA2365	**Hypothetical, unclassified, unknown**	-4.81	-10.73		-5.46			COG3516, Uncharacterized protein conserved in bacteria [Function unknown].
PA14_10350	PA2366	**Hypothetical, unclassified, unknown**	-3.52	-3.89		-6.29	-3.13		COG3517, Uncharacterized protein conserved in bacteria [Function unknown].
PA14_26990	PA2368	**Hypothetical, unclassified, unknown**	-2.84	-2.44		-11.89	-3.69		COG3518, Uncharacterized protein conserved in bacteria [Function unknown].

PA14_29020	PA2381	**Hypothetical, unclassified, unknown**		-2.41	-2.18		-3.09			hypothetical protein
PA14_17480	PA2405	**Hypothetical, unclassified, unknown**		-2.08	-7.69		-12.61	3.54	-2.78	hypothetical protein
PA14_39860	PA2406	**Hypothetical, unclassified, unknown**		-2.06	-2.34	3.35	-3.06			
PA14_33250	PA2427	**Hypothetical, unclassified, unknown**			-9.59	5.91			-10.80	hypothetical protein
PA14_33160	PA2433	**Hypothetical, unclassified, unknown**			-4.60	2.22	-5.13			hypothetical protein
PA14_03510	PA2448	**Hypothetical, unclassified, unknown**		-3.21	-5.24	4.88	-3.45	-3.40		COG1574, Predicted metal-dependent hydrolase with the TIM-barrel fold [General function prediction only].
PA14_32280	PA2504	**Hypothetical, unclassified, unknown**			-4.44	3.82	-3.83	-3.08		COG4859, Uncharacterized protein conserved in bacteria [Function unknown].
PA14_46780	PA2544	**Hypothetical, unclassified, unknown**		-2.17	-2.32					RimI, Acetyltransferases [General function prediction only].
PA14_70940	PA2564	**Hypothetical, unclassified, unknown**	*tam*	-3.94	-2.56	4.94				Tam, Trans-aconitate methyltransferase [General function prediction only].
PA14_70950	PA2565	**Hypothetical, unclassified, unknown**		-2.30	-2.32	6.14		-3.98		hypothetical protein
PA14_36320	PA2566	**Hypothetical, unclassified, unknown**		-2.64	-2.38	4.04	-4.00			HcaD, Uncharacterized NAD(FAD)-dependent dehydrogenases [General function prediction only].
PA14_15120	PA2658	**Hypothetical, unclassified, unknown**		-2.06	-2.31					COG3212, Predicted membrane protein [Function unknown].
PA14_40250	PA2659	**Hypothetical, unclassified, unknown**		-2.95	-2.00	-2.31				COG3212, Predicted membrane protein [Function unknown].
PA14_28600	PA2747	**Hypothetical, unclassified, unknown**		-2.22	-2.59					
PA14_05620	PA2927	**Hypothetical, unclassified, unknown**		-2.45	-2.59		-3.70			hypothetical protein
PA14_44290	PA3091	**Hypothetical, unclassified, unknown**		-2.07	-2.21					Smc, Chromosome segregation ATPases [Cell division and chromosome partitioning].
PA14_22320	PA3237	**Hypothetical, unclassified, unknown**		-92.82	-81.97	56.49		-14.81	-5.94	COG3205, Predicted membrane protein [Function unknown].
PA14_36630	PA3274	**Hypothetical, unclassified, unknown**		-3.13	-2.68	3.45				hypothetical protein
PA14_10360	PA3287	**Hypothetical, unclassified, unknown**		-6.46	-15.82	13.81		-19.31		Arp, FOG: Ankyrin repeat [General function prediction only].

PA14_20460	PA3371	**Hypothetical, unclassified, unknown**			-5.76	4.08		-4.65	-3.09	hypothetical protein
PA14_19110	PA3520	**Hypothetical, unclassified, unknown**		-10.12	-5.36	9.66			-2.41	CopZ, Copper chaperone [Inorganic ion transport and metabolism].
PA14_18680	PA3530	**Hypothetical, unclassified, unknown**	*bfd*	-3.17	-7.30	8.97				Bfd, Bacterioferritin-associated ferredoxin [Inorganic ion transport and metabolism].
PA14_16640	PA3691	**Hypothetical, unclassified, unknown**			-8.50	3.19	-3.04		-2.42	hypothetical protein
PA14_40240	PA3734	**Hypothetical, unclassified, unknown**		-2.70	-2.44					
PA14_50810	PA3784	**Hypothetical, unclassified, unknown**		-2.29	-4.79	-2.91	-7.58	-4.24		hypothetical protein
PA14_33870	PA3785	**Hypothetical, unclassified, unknown**		-2.19	-2.38	-2.28	-3.56	-6.21		COG2847, Uncharacterized protein conserved in bacteria [Function unknown].
PA14_14740	PA3815	**Hypothetical, unclassified, unknown**		-5.28	-3.46	5.68	4.35			COG1959, Predicted transcriptional regulator [Transcription].
PA14_14290	PA3844	**Hypothetical, unclassified, unknown**			-2.09	2.69				Gst, Glutathione S-transferase [Posttranslational modification, protein turnover, chaperones].
PA14_05580	PA3923	**Hypothetical, unclassified, unknown**		-2.16	-4.53	-5.49	-4.67	-3.92	-2.46	hypothetical protein
PA14_13050	PA3928	**Hypothetical, unclassified, unknown**		-3.28	-2.71	4.68				hypothetical protein
PA14_10380	PA4139	**Hypothetical, unclassified, unknown**		-2.08	-7.09		-3.91			
PA14_31290	PA4141	**Hypothetical, unclassified, unknown**		-5.04	-2.21	9.94				hypothetical protein
PA14_20470	PA4220	**Hypothetical, unclassified, unknown**	*fptB*	-53.20	-7.75	5.18	-7.19	-5.35	-4.58	hypothetical protein
PA14_55840	PA4298	**Hypothetical, unclassified, unknown**			-2.61	4.04	-6.21			hypothetical protein
PA14_14730	PA4384	**Hypothetical, unclassified, unknown**		-4.14	-3.28	4.46				COG4318, Uncharacterized protein conserved in bacteria [Function unknown].
PA14_58010	PA4469	**Hypothetical, unclassified, unknown**			-110.25	4.28	-3.61		-3.60	hypothetical protein
PA14_09490	PA4573	**Hypothetical, unclassified, unknown**		-2.42	-3.38					hypothetical protein

PA14_61550	PA4607	**Hypothetical, unclassified, unknown**		-2.35	-23.67		-5.49		2.17	hypothetical protein
PA14_65040	PA4624	**Hypothetical, unclassified, unknown**		-2.29	-37.78					FhaC, Hemolysin activation/secretion protein [Intracellular trafficking and secretion].
PA14_13340	PA4625	**Hypothetical, unclassified, unknown**		-2.51	-3.42	3.39				hypothetical protein
PA14_40100	PA4641	**Hypothetical, unclassified, unknown**		-2.49	-2.21					still frameshift hypothetical protein
PA14_61430	PA4642	**Hypothetical, unclassified, unknown**		-4.13	5.38		5.83	3.02		hypothetical protein
PA14_43050	PA4648	**Hypothetical, unclassified, unknown**		-2.09	-2.24		-3.52			COG5430, Uncharacterized secreted protein [Function unknown].
PA14_32950	PA4650	**Hypothetical, unclassified, unknown**		-2.32	-5.10	5.26			-2.28	COG5430, Uncharacterized secreted protein [Function unknown].
PA14_61540	PA4652	**Hypothetical, unclassified, unknown**			-9.23	6.55				FimD, P pilus assembly protein, porin PapC [Cell motility and secretion / Intracellular trafficking and secretion].
PA14_09400	PA4653	**Hypothetical, unclassified, unknown**		-2.76	-4.79		-4.13			COG5430, Uncharacterized secreted protein [Function unknown].
PA14_62680	PA4738	**Hypothetical, unclassified, unknown**	*yjbJ*		-4.98	9.47		-4.90	-3.40	COG3237, Uncharacterized protein conserved in bacteria [Function unknown].
PA14_63330	PA4792	**Hypothetical, unclassified, unknown**			-2.79	2.20				UgpQ, Glycerophosphoryl diester phosphodiesterase [Energy production and conversion].
PA14_60520	PA4874	**Hypothetical, unclassified, unknown**	*psiF*	-2.20	-2.52		-4.69			conserved hypothetical protein
PA14_64930	PA4916	**Hypothetical, unclassified, unknown**		-2.05	-3.18					COG1051, ADP-ribose pyrophosphatase [Nucleotide transport and metabolism].
PA14_34030	PA4925	**Hypothetical, unclassified, unknown**		-2.60	-2.65		-3.22			MscS, Small-conductance mechanosensitive channel [Cell envelope biogenesis, outer membrane].
PA14_55850	PA5023	**Hypothetical, unclassified, unknown**	*ydiU*	-2.72	-37.36	3.96				COG0397, Uncharacterized conserved protein [Function unknown].
PA14_41500	PA5061	**Hypothetical, unclassified, unknown**	*phaI*	-2.31	-2.87					conserved hypothetical protein
PA14_72520	PA5220	**Hypothetical, unclassified, unknown**		-2.13	-2.11					hypothetical protein
PA14_69640	PA5275	**Hypothetical, unclassified, unknown**	*cyaY*		-13.55	4.21	4.34		2.98	CyaY, Protein implicated in iron transport, frataxin homolog [Inorganic ion transport and metabolism].

PA14_71100	PA5383	**Hypothetical, unclassified, unknown**	*yeiH*			7.82		-6.54	-3.66	COG2855, Predicted membrane protein [Function unknown].
PA14_71200	PA5392	**Hypothetical, unclassified, unknown**		-3.35	-3.81					TdcF, Putative translation initiation inhibitor, yjgF family [Translation, ribosomal structure and biogenesis].
PA14_10330	PA5395	**Hypothetical, unclassified, unknown**		-2.11	-3.62				2.74	COG3558, Uncharacterized protein conserved in bacteria [Function unknown].
PA14_08210	PA4299	**Motility and Attachment**	*tadD*	-2.57	-2.34	3.84	-4.31			hypothetical protein
PA14_33480	PA4303	**Motility and Attachment**	*tadZ*	-2.95	-11.11	5.97	-3.83			hypothetical protein
PA14_55930	PA4305	**Motility and Attachment**	*rcpC*		-2.26	2.73				hypothetical protein
PA14_08260	PA4306	**Motility and Attachment**	*flp*	-4.09	-15.54	3.31			2.08	Flp, Flp pilus assembly protein, pilin Flp [Intracellular trafficking and secretion].
PA14_34880	PA4651	**Motility and Attachment**		-2.62	-14.19		-14.81			FimC, P pilus assembly protein, chaperone PapD [Cell motility and secretion / Intracellular trafficking and secretion].
PA14_61020	PA0849	**Nucleotide biosynthesis**	*trxB2*	-4.62	-6.49	10.97		-8.13	-3.39	TrxB, Thioredoxin reductase [Posttranslational modification, protein turnover, chaperones].
PA14_61530	PA5496	**Nucleotide biosynthesis**	*nrdJb*	-2.31	-10.67					hypothetical protein
PA14_13030	PA5497	**Nucleotide biosynthesis**	*nrdJa*	-3.13	-8.74					hypothetical protein
PA14_48620	PA1875	**Protein secretion/export apparatus**	*opmL*	-3.73	-5.08		-12.55		-2.19	TolC, Outer membrane protein [Cell envelope biogenesis, outer membrane / Intracellular trafficking and secretion].
PA14_21670	PA1877	**Protein secretion/export apparatus**		-2.59	-3.56	3.83	-14.43			EmrA, Multidrug resistance efflux pump [Defense mechanisms].
PA14_09380	PA4142	**Protein secretion/export apparatus**		-4.62	-5.11					EmrA, Multidrug resistance efflux pump [Defense mechanisms].
PA14_61410	PA4144	**Protein secretion/export apparatus**	*opmK*	-2.25	-539.10					TolC, Outer membrane protein [Cell envelope biogenesis, outer membrane / Intracellular trafficking and secretion].
PA14_08120	PA4302	**Protein secretion/export apparatus**	*tadA, hvbA*	-2.20	-2.43	3.28	-22.42		-5.12	probable type II secretion system protein, CpaF, Flp pilus assembly protein, ATPase CpaF [Intracellular trafficking and secretion].
PA14_34070	PA4304	**Protein secretion/export apparatus**	*rcpA, xqhC*	-3.64	-6.78		-3.16			probable type II secretion system protein
PA14_15130	PA0249	**Putative enzymes**		-2.11	-2.71					RimI, Acetyltransferases [General function prediction only].
PA14_34020	PA0704	**Putative enzymes**		-2.44	-12.00		-10.08			GatA, Asp-tRNAAsn/Glu-tRNAGln amidotransferase A subunit and related amidases [Translation, ribosomal structure and biogenesis].
PA14_53110	PA0863	**Putative enzymes**			-2.21	3.82		-3.89	-2.24	Qor, NADPH:quinone reductase and related Zn-dependent oxidoreductases [Energy production and conversion / General function prediction only].
PA14_51050	PA1027	**Putative enzymes**	*pcd*		-2.79	3.06				PutA, NAD-dependent aldehyde dehydrogenases [Energy production and conversion].

PA14_09900	PA1240	**Putative enzymes**		-2.85	-6.54					CaiD, Enoyl-CoA hydratase/carnithine racemase [Lipid metabolism].
PA14_47550	PA1287	**Putative enzymes**		-2.55	-2.34					BtuE, Glutathione peroxidase [Posttranslational modification, protein turnover, chaperones].
PA14_66160	PA1391	**Putative enzymes**				23.82		-7.19	-3.48	RfaG, Glycosyltransferase [Cell envelope biogenesis, outer membrane].
PA14_42980	PA1662	**Putative enzymes**		-2.15	-4.25					ClpA, ATPases with chaperone activity, ATP-binding subunit [Posttranslational modification, protein turnover, chaperones].
PA14_40430	PA1860	**Putative enzymes**			-2.12	2.34				COG3315, O-Methyltransferase involved in polyketide biosynthesis [Secondary metabolites biosynthesis, transport, and catabolism].
PA14_19920	PA1880	**Putative enzymes**		-2.20	-11.64		-4.50			CoxL, Aerobic-type carbon monoxide dehydrogenase, large subunit CoxL/CutL homologs [Energy production and conversion].
PA14_27730	PA1881	**Putative enzymes**		-2.13	-5.49		-5.29			CoxS, Aerobic-type carbon monoxide dehydrogenase, small subunit CoxS/CutS homologs [Energy production and conversion].
PA14_40200	PA2067	**Putative enzymes**		-2.08	-7.21		-4.63			COG0637, Predicted phosphatase/phosphohexomutase [General function prediction only].
PA14_36490	PA2069	**Putative enzymes**		-3.41	-2.53	7.44	-3.83			COG2192, Predicted carbamoyl transferase, NodU family [Posttranslational modification, protein turnover, chaperones].
PA14_37340	PA2108	**Putative enzymes**				3.53		-3.03	-3.27	IlvB, Thiamine pyrophosphate-requiring enzymes [acetolactate synthase, pyruvate dehydrogenase (cytochrome), glyoxylate carboligase, phosphonopyruvate decarboxylase] [Amino acid transport and metabolism / Coenzyme metabolism].
PA14_55890	PA2152	**Putative enzymes**		-2.92	-5.45	3.37	-4.81		-2.50	Ble, Uncharacterized protein, probably involved in trehalose biosynthesis [Carbohydrate transport and metabolism].
PA14_40260	PA2158	**Putative enzymes**		-7.91	-3.41	4.58	-13.76	-3.14	-6.93	Tdh, Threonine dehydrogenase and related Zn-dependent dehydrogenases [Amino acid transport and metabolism / General function prediction only].
PA14_01710	PA2160	**Putative enzymes**	*glgX*	-10.06	-5.83	3.33	-11.72		-4.66	PulA, Type II secretory pathway, pullulanase PulA and related glycosidases [Carbohydrate transport and metabolism].
PA14_36590	PA2162	**Putative enzymes**		-2.27	-6.80	4.10	-5.10		-2.58	TreY, Maltooligosyl trehalose synthase [Carbohydrate transport and metabolism].
PA14_36580	PA2164	**Putative enzymes**			-8.33	4.98	-3.55			GlgB, 1,4-alpha-glucan branching enzyme [Carbohydrate transport and metabolism].
PA14_35150	PA2275	**Putative enzymes**	*yahK*			12.10	5.88	-5.65	-3.77	AdhP, Zn-dependent alcohol dehydrogenases [General function prediction only].
PA14_31660	PA2298	**Putative enzymes**		-2.05	-43.31					SdhA, Succinate dehydrogenase/fumarate reductase, flavoprotein subunit [Energy production and conversion].
PA14_47190	PA2378	**Putative enzymes**		-3.65	-2.26		-3.83			CoxL, Aerobic-type carbon monoxide dehydrogenase, large subunit CoxL/CutL homologs [Energy production and conversion].
PA14_16100	PA2815	**Putative enzymes**	*yafH*	-2.31	-10.38		-5.21			CaiA, Acyl-CoA dehydrogenases [Lipid metabolism].
PA14_19360	PA3460	**Putative enzymes**		-2.32	-9.03	7.14	-6.17	-7.58	-6.30	DdlA, D-alanine-D-alanine ligase and related ATP-grasp enzymes [Cell envelope biogenesis, outer membrane].

PA14_16930	PA3667	**Putative enzymes**		-2.76	-2.17					CsdB, Selenocysteine lyase [Amino acid transport and metabolism].
PA14_12680	PA3957	**Putative enzymes**		-2.10	-9.65	3.42			2.78	DltE, Short-chain dehydrogenases of various substrate specificities [General function prediction only].
PA14_34520	PA4079	**Putative enzymes**		-2.77	-4.53					DltE, Short-chain dehydrogenases of various substrate specificities [General function prediction only].
PA14_09630	PA4199	**Putative enzymes**		-2.09	-2.28					CaiA, Acyl-CoA dehydrogenases [Lipid metabolism].
PA14_62330	PA4709	**Putative enzymes**	*phuS*	-2.14	-5.46	5.36			-2.83	HemS, Putative heme degradation protein [Inorganic ior transport and metabolism].
PA14_62400	PA4715	**Putative enzymes**	*yfdZ*	-2.20	-4.69				-2.02	COG0436, Aspartate/tyrosine/aromatic aminotransferase [Amino acid transport and metabolism].
PA14_68040	PA5150	**Putative enzymes**			-2.51	4.38				FabG, Dehydrogenases with different specificities (related to short-chain alcohol dehydrogenases) [Secondary metabolites biosynthesis, transport, and catabolism / General function prediction only].
PA14_36650	PA5181	**Putative enzymes**		-2.50	-12.82	4.76				BisC, Anaerobic dehydrogenases, typically selenocysteine-containing [Energy production and conversion].
PA14_70160	PA5313	**Putative enzymes**	*paaT*		-2.39	4.89			-3.10	BioA, Adenosylmethionine-8-amino-7-oxononanoate aminotransferase [Coenzyme metabolism].
PA14_71110	PA5384	**Putative enzymes**			-2.23	5.21		-5.62	-6.31	Aes, Esterase/lipase [Lipid metabolism].
PA14_73140	PA5546	**Putative enzymes**			-11.81	9.44		-4.42		Cfa, Cyclopropane fatty acid synthase and related methyltransferases [Cell envelope biogenesis, outer membrane].
PA14_48980	PA0719	**Related to phage, transposon, or plasmid**			-22.17	2.53				hypothetical protein of bacteriophage Pf1
PA14_03010	PA0243	**Transcriptional regulators**		-2.85	-2.26					AcrR, Transcriptional regulator [Transcription].
PA14_06180	PA0472	**Transcriptional regulators**	*fiuI*		-4.42	7.00		-3.88	-2.53	probable sigma-70 factor, ECF subfamily
PA14_55550	PA0675	**Transcriptional regulators**			-3.24	6.46		-3.46	-8.73	RpoE, DNA-directed RNA polymerase specialized sigma subunit, sigma24 homolog [Transcription].
PA14_08010	PA1300	**Transcriptional regulators**		-2.21	-4.24	3.13	4.45		-2.79	RpoE, DNA-directed RNA polymerase specialized sigma subunit, sigma24 homolog [Transcription].
PA14_48640	PA2196	**Transcriptional regulators**		-4.78	-2.27			3.92	2.88	AcrR, Transcriptional regulator [Transcription].
PA14_35540	PA2246	**Transcriptional regulators**	*bkdR*	-2.14	-2.24	2.03				Lrp, Transcriptional regulators [Transcription].
PA14_36710	PA2299	**Transcriptional regulators**		-2.96	-5.48					PhnF, Transcriptional regulators [Transcription].
PA14_33260	PA2426	**Transcriptional regulators**	*pvdS*		-33.33	48.69	-3.85		-30.93	sigma factor PvdS
PA14_32710	PA2468	**Transcriptional regulators**	*foxI*		-11.04	4.75		-4.98	-3.40	probable sigma-70 factor, ECF subfamily
PA14_36605	PA2588	**Transcriptional regulators**		-2.36	-2.90	4.29		-3.25		AraC, AraC-type DNA-binding domain-containing proteins, probable transcriptional regulator [Transcription].
PA14_26190	PA3622	**Transcriptional regulators**	*rpoS*	-2.23	-9.31		-5.35			RpoD, DNA-directed RNA polymerase, sigma subunit (sigma70/sigma32) [Transcription].

PA14_36820	PA5059	**Transcriptional regulators**	*phaD*	-3.25	-2.84	8.29	-6.13			AcrR, Transcriptional regulator [Transcription].
	Pae_tR	**Translation, post-translational modification, degradation**		-5.36	-3.10	5.22	-6.13	-11.15	-12.12	tRNA_Cysteine, 2918603-2918676 (-) strand
PA14_49130	PA1183	**Transport of small molecules**	*dctA*	-2.35	-10.03					GltP, Na+/H+-dicarboxylate symporters [Energy production and conversion].
PA14_47160	PA2329	**Transport of small molecules**		-4.51	-4.07		-5.99			TauB, ABC-type nitrate/sulfonate/bicarbonate transport system, ATPase component [Inorganic ion transport and metabolism].
PA14_34970	PA3186	**Transport of small molecules**	*oprB*	-2.48	-2.34				2.68	OprB, Carbohydrate-selective porin [Cell envelope biogenesis, outer membrane].
PA14_55860	PA3187	**Transport of small molecules**	*gltK*	-2.04	-2.52		-3.30			MalK, ABC-type sugar transport systems, ATPase components [Carbohydrate transport and metabolism].
PA14_37780	PA3189	**Transport of small molecules**	*gltF*	-2.52	-2.85					UgpA, ABC-type sugar transport systems, permease components [Carbohydrate transport and metabolism].
PA14_66410	PA3190	**Transport of small molecules**	*gltB*	-2.41	-2.49	4.11				UgpB, ABC-type sugar transport system, periplasmic component [Carbohydrate transport and metabolism].
PA14_20450	PA3372	**Transport of small molecules**	*phnP*			8.05		-4.10	-3.43	PhnP, Metal-dependent hydrolases of the beta-lactamase superfamily I [General function prediction only].
PA14_20010	PA3408	**Transport of small molecules**	*hasR*		-2.27	3.35			-2.05	heme acquisition protein HasR
PA14_15070	PA3790	**Transport of small molecules**	*oprC*	-2.04	-4.22					CirA, Outer membrane receptor proteins, mostly Fe transport [Inorganic ion transport and metabolism].
PA14_11140	PA4206	**Transport of small molecules**	*mexH*	-71.64	-151.80	47.32	-3.14			RND efflux , AcrA, Membrane-fusion protein [Cell envelope biogenesis, outer membrane].
PA14_06170	PA0471	**Two-component regulatory systems**	*fiuR*	-2.88	-5.29	4.76				FecR, Fe2+-dicitrate sensor, membrane component [Inorganic ion transport and metabolism / Signal transduction mechanisms].
PA14_29360	PA2687	**Two-component regulatory systems**	*pfeS*		-3.62	4.69		-3.27	-3.75	BaeS, Signal transduction histidine kinase [Signal transduction mechanisms].
PA14_30240	PA4293	**Two-component regulatory systems**	*pprA*	-2.00	-2.62	2.26	-6.67			COG4191, Signal transduction histidine kinase regulating C4-dicarboxylate transport system [Signal transduction mechanisms].

Negatively regulated genes

PA14_43220	PA1646	**Adaptation, Protection**			2.23	-6.33		4.09	2.62	Tar, Methyl-accepting chemotaxis protein [Cell motility and secretion / Signal transduction mechanisms].
PA14_21020	PA3327	**Adaptation, Protection**			5.62	-3.50				EntF, Non-ribosomal peptide synthetase modules and related proteins [Secondary metabolites biosynthesis, transport, and catabolism].
PA14_20950	PA3331	**Adaptation, Protection**		2.55	4.30	-3.52				CypX, Cytochrome P450 [Secondary metabolites biosynthesis, transport, and catabolism].
PA14_18360	PA3553	**Adaptation, Protection**	*arnC, pmrF*	2.52	2.18	-6.90	-8.93			WcaA, Glycosyltransferases involved in cell wall biogenesis [Cell envelope biogenesis, outer membrane].

PA14_61780	PA4671	**Adaptation, Protection**	*rplY*	2.05	3.52					RplY, Ribosomal protein L25 (general stress protein Ctc) [Translation, ribosomal structure and biogenesis].
PA14_06750	PA0132	**Amino acid biosynthesis**	*oapT*	3.00	3.41	-5.41		3.98	3.11	beta-alanine--pyruvate transaminase
PA14_51330	PA1004	**Amino acid biosynthesis**	*nadA*		2.03	-3.28				NadA, Quinolinate synthase [Coenzyme metabolism].
PA14_44010	PA1585	**Amino acid biosynthesis**	*sucA*		-2.56	-2.83	-3.98	-4.24	-2.21	SucA, 2-oxoglutarate dehydrogenase complex, dehydrogenase (E1) component, and related enzymes [Energy production and conversion].
PA14_35530	PA2247	**Amino acid biosynthesis**	*bkdA1*		-4.83	-3.56	-7.09	-4.41	-2.51	2-oxoisovalerate dehydrogenase (alpha subunit)
PA14_62600	PA4731	**Amino acid biosynthesis**	*panD*		2.11	-3.08				PanD, Aspartate 1-decarboxylase [Coenzyme metabolism].
PA14_12440	PA4759	**Amino acid biosynthesis**	*dapB*	4.93	3.02	-4.57				DapB, Dihydrodipicolinate reductase [Amino acid transport and metabolism].
PA14_65110	PA4930	**Amino acid biosynthesis**	*alr*		7.13	-3.03	3.93	4.33	2.49	Alr, Alanine racemase [Cell envelope biogenesis, outer membrane].
PA14_65795	PA4977	**Amino acid biosynthesis**	*aruI*	2.00	4.58					IlvB, Thiamine pyrophosphate-requiring enzymes [acetolactate synthase, pyruvate dehydrogenase (cytochrome), glyoxylate carboligase, phosphonopyruvate decarboxylase] [Amino acid transport and metabolism / Coenzyme metabolism].
PA14_11690	PA5495	**Amino acid biosynthesis**	*thrB*	6.25	2.15	-2.55				COG2334, Putative homoserine kinase type II (protein kinase fold) [General function prediction only].
PA14_38580	PA0509	**Biosynthesis of cofactors**	*nirN*	2.06	6.83					CccA, Cytochrome c, mono- and diheme variants [Energy production and conversion].
PA14_34900	PA0510	**Biosynthesis of cofactors**	*nirE*	5.26	18.31					CysG, Uroporphyrinogen-III methylase [Coenzyme metabolism].
PA14_37770	PA0511	**Biosynthesis of cofactors**	*nirJ*	2.99	2.63					COG0535, Predicted Fe-S oxidoreductases [General function prediction only].
PA14_09230	PA0513	**Biosynthesis of cofactors**	*nirG*	3.36	8.65					Lrp, Transcriptional regulators [Transcription].
PA14_29720	PA0515	**Biosynthesis of cofactors**	*nirD*	3.36	2.38					Lrp, Transcriptional regulators [Transcription].
PA14_60700	PA0517	**Biosynthesis of cofactors**	*nirC*	5.21	28.40	-6.17				CccA, Cytochrome c, mono- and diheme variants [Energy production and conversion].
PA14_72260	PA0518	**Biosynthesis of cofactors**	*nirM*	5.65	13.90	-4.03			2.08	cytochrome c-551 precursor
PA14_44470	PA1546	**Biosynthesis of cofactors**	*hemN*	2.19	8.48	-4.05				HemN, Coproporphyrinogen III oxidase and related Fe-S oxidoreductases [Coenzyme metabolism].
PA14_13260	PA3915	**Biosynthesis of cofactors**	*moaB1*	23.26	45.13	-3.36				MoaB, Molybdopterin biosynthesis enzymes [Coenzyme metabolism].
PA14_12020	PA4006	**Biosynthesis of cofactors**	*nadD, ybeN*		3.05	-3.21				NadD, Nicotinic acid mononucleotide adenylyltransferase [Coenzyme metabolism].
PA14_28050	PA4919	**Biosynthesis of cofactors**	*pncB1*	2.34	2.15			4.93		PncB, Nicotinic acid phosphoribosyltransferase [Coenzyme metabolism].
PA14_02550	PA0208	**Carbon compound catabolism**	*mdcA*	2.56		-4.55	5.62	7.01	4.81	malonate decarboxylase alpha subunit
PA14_02610	PA0213	**Carbon compound catabolism**	*mdcG*	3.19	2.08		5.61			hypothetical protein

PA14_54000	PA0792	**Carbon compound catabolism**	*prpD*		-4.01	-2.53	-6.90			PrpD, Uncharacterized protein involved in propionate catabolism [General function prediction only].
PA14_70390	PA5332	**Carbon compound catabolism**	*crc*		2.26	-4.02	3.95	3.96	3.76	catabolite repression control protein
PA14_64950	PA3337	**Cell wall / LPS / capsule**	*rfaD*	7.19	9.58	-5.03		4.44	6.06	ADP-L-glycero-D-mannoheptose 6-epimerase
PA14_72500	PA3584	**Central intermediary metabolism**	*glpD*	2.00	-4.07	-5.65				GlpA, Glycerol-3-phosphate dehydrogenase [Energy production and conversion].
PA14_43770	PA4563	**Central intermediary metabolism**	*rpsT*	2.35	2.44	-2.99	30.05		2.01	RpsT, Ribosomal protein S20 [Translation, ribosomal structure and biogenesis].
PA14_62830	PA4748	**Central intermediary metabolism**	*tpiA*		5.78	-3.11	5.05	5.17	2.50	TpiA, Triosephosphate isomerase [Carbohydrate transport and metabolism].
PA14_71740	PA5436	**Central intermediary metabolism**		3.42	5.03	-7.52				AccC, Biotin carboxylase [Lipid metabolism].
PA14_23680	PA3126	**Chaperones and heat shock proteins**	*ibpA, hslT*	4.78	2.65	-6.85			2.22	IbpA, Molecular chaperone, heat shock protein (small heat shock protein) [Posttranslational modification, protein turnover, chaperones].
PA14_61000	PA5053	**Chaperones and heat shock proteins**	*hslV*	3.97	3.97	-3.50				HslV, ATP-dependent protease HslVU (ClpYQ), peptidase subunit [Posttranslational modification, protein turnover, chaperones].
PA14_07530	PA0577	**DNA replication, recombination, modification and**	*dnaG*		4.98	-3.17				DnaG, DNA primase (bacterial type) [DNA replication, recombination, and repair].
PA14_15970	PA3745	**DNA replication, recombination, modification and repair**	*rpsP*		8.62	-3.03	3.50	3.16	2.29	RpsP, Ribosomal protein S16 [Translation, ribosomal structure and biogenesis].
PA14_04750	PA0362	**Energy metabolism**	*fdx1*		3.00	-3.19				ferredoxin [4Fe-4S]
PA14_66770	PA0516	**Energy metabolism**	*nirF*	2.79	5.96	-3.42				heme d1 biosynthesis protein NirF
PA14_21010	PA0519	**Energy metabolism**	*nirS*	4.69	53.09	-5.56				nitrite reductase precursor
PA14_06810	PA0523	**Energy metabolism**	*norC*	5.41	24.76					nitric-oxide reductase subunit C
PA14_44360	PA1555	**Energy metabolism**	*ccoP, fixP*	2.96	6.44	-9.52			3.44	CccA, Cytochrome c, mono- and diheme variants [Energy production and conversion].
PA14_44350	PA1556	**Energy metabolism**	*ccoO, fixO*	2.77	9.35	-8.47			3.13	CcoO, Cbb3-type cytochrome oxidase, cytochrome c subunit [Energy production and conversion].
PA14_44340	PA1557	**Energy metabolism**	*ccoN, fixN, cytN*	2.83	27.46	-12.21			2.79	CcoN, Cbb3-type cytochrome oxidase, subunit 1 [Posttranslational modification, protein turnover, chaperones].
PA14_34640	PA2321	**Energy metabolism**	*gntV, gntK, gnuK*		3.62	-3.91	3.13	8.87	5.41	gluconokinase
PA14_32530	PA2482	**Energy metabolism**		2.04	2.18					COG2863, Cytochrome c553 [Energy production and conversion].
PA14_01620	PA3621	**Energy metabolism**	*fdxA*	3.86	13.28	-5.26	5.79		2.08	COG1146, Ferredoxin [Energy production and conversion].
PA14_13830	PA3872	**Energy metabolism**	*narI*	2.28	2.50					NarI, Nitrate reductase gamma subunit [Energy production and conversion].

PA14_13810	PA3873	**Energy metabolism**	*narJ*	2.58	2.03					NarJ, Nitrate reductase delta subunit [Energy production and conversion].
PA14_13740	PA3878	**Energy metabolism**	*narX*		5.03	-6.58				NarQ, Signal transduction histidine kinase, nitrate/nitrite-specific [Signal transduction mechanisms].
PA14_13730	PA3879	**Energy metabolism**	*narL*	5.41	4.67	-8.06			3.16	two-component response regulator , CitB, Response regulator containing a CheY-like receiver domain and an HTH DNA-binding domain [Signal transduction mechanisms / Transcription].
PA14_09030	PA4571	**Energy metabolism**		2.15	9.25	-3.38				COG3258, Cytochrome c [Energy production and conversion].
PA14_30100	PA5427	**Energy metabolism**	*adhA*	3.29	2.56	-3.82			3.08	AdhP, Zn-dependent alcohol dehydrogenases [General function prediction only].
PA14_20980	PA3333	**Fatty acid and phospholipid metabolism**	*fabH2*	2.56	6.69	-3.76	-4.81			FabH, 3-oxoacyl-[acyl-carrier-protein].
PA14_20890	PA3334	**Fatty acid and phospholipid metabolism**		3.75	3.97	-4.44				hypothetical protein
PA14_17675	PA3603	**Fatty acid and phospholipid metabolism**	*dgkA*		2.83	-3.98		3.72	2.25	DgkA, Diacylglycerol kinase [Cell envelope biogenesis, outer membrane].
PA14_66080	PA4997	**Fatty acid and phospholipid metabolism**	*msbA*		5.46	-3.14				MdlB, ABC-type multidrug transport system, ATPase and permease components [Defense mechanisms].
PA14_01730	PA0141	**Hypothetical, unclassified, unknown**		2.19	4.58	-6.85				COG2326, Uncharacterized conserved protein [Function unknown].
PA14_02060	PA0165	**Hypothetical, unclassified, unknown**		2.03	2.88		3.18			Tsx, Nucleoside-binding outer membrane protein [Cell envelope biogenesis, outer membrane].
PA14_02530	PA0201	**Hypothetical, unclassified, unknown**		3.46	21.46	-9.35	53.47	8.21	12.12	COG3545, Predicted esterase of the alpha/beta hydrolase fold [General function prediction only].
PA14_03160	PA0256	**Hypothetical, unclassified, unknown**				-5.38		4.59	2.20	hypothetical protein
PA14_06860	PA0526	**Hypothetical, unclassified, unknown**		4.07	14.62					hypothetical protein
PA14_06890	PA0529	**Hypothetical, unclassified, unknown**		5.59	2.37	-3.36				COG2258, Uncharacterized protein conserved in bacteria [Function unknown].
PA14_07040	PA0542	**Hypothetical, unclassified, unknown**	*yqjC*	2.34	2.53					conserved hypothetical protein
PA14_07330	PA0563	**Hypothetical, unclassified, unknown**		2.21	3.62		8.94		2.04	COG3152, Predicted membrane protein [Function unknown].
PA14_54820	PA0731	**Hypothetical, unclassified, unknown**		2.25	3.42					hypothetical protein
PA14_51850	PA0960	**Hypothetical, unclassified, unknown**				-3.02		3.27	2.80	SlyX, Uncharacterized protein conserved in bacteria [Function unknown].

PA14_51690	PA0974	**Hypothetical, unclassified, unknown**			2.22	-5.03				COG1729, Uncharacterized protein conserved in bacteria [Function unknown].
PA14_48870	PA1192	**Hypothetical, unclassified, unknown**	*ydaO*		7.25	-3.34	3.19			MesJ, Predicted ATPase of the PP-loop superfamily implicated in cell cycle control [Cell division and chromosome partitioning].
PA14_20940	PA1343	**Hypothetical, unclassified, unknown**		6.80	2.01	-4.29		-5.88	-4.12	hypothetical protein
PA14_44270	PA1564	**Hypothetical, unclassified, unknown**		3.38	2.46					SirA, Predicted redox protein, regulator of disulfide bond formation [Posttranslational modification, protein turnover, chaperones].
PA14_44140	PA1574	**Hypothetical, unclassified, unknown**	*yaiE*	3.75	4.48	-3.53				COG3123, Uncharacterized protein conserved in bacteria [Function unknown].
PA14_23010	PA1604	**Hypothetical, unclassified, unknown**		5.52	2.27					HisJ, ABC-type amino acid transport/signal transduction systems, periplasmic component/domain [Amino acid transport and metabolism / Signal transduction mechanisms].
PA14_43720	PA1607	**Hypothetical, unclassified, unknown**			2.07	-4.85		3.22	3.51	COG1733, Predicted transcriptional regulators [Transcription].
PA14_42860	PA1673	**Hypothetical, unclassified, unknown**		4.27	10.49	-39.06	6.45	12.71	17.79	COG2703, Hemerythrin [Inorganic ion transport and metabolism].
PA14_60490	PA1746	**Hypothetical, unclassified, unknown**		4.18	4.16	-3.31		4.31	6.58	COG2110, Predicted phosphatase homologous to the C terminal domain of histone macroH2A1 [General function prediction only].
PA14_41440	PA1789	**Hypothetical, unclassified, unknown**		2.33	-3.11	-7.58	-3.73		2.50	UspA, Universal stress protein UspA and related nucleotide-binding proteins [Signal transduction mechanisms].
PA14_41030	PA1817	**Hypothetical, unclassified, unknown**		2.42	-6.09	-5.46		3.24	4.10	COG3631, Ketosteroid isomerase-related protein [General function prediction only].
PA14_37080	PA2126	**Hypothetical, unclassified, unknown**		2.47	3.04					Spo0J, Predicted transcriptional regulators [Transcription].
PA14_37070	PA2127	**Hypothetical, unclassified, unknown**		8.40	4.85	-9.17	6.20			COG3969, Predicted phosphoadenosine phosphosulfate sulfotransferase [General function prediction only].
PA14_36450	PA2176	**Hypothetical, unclassified, unknown**		2.10	-4.36	2.99				COG2910, Putative NADH-flavin reductase [General function prediction only].
PA14_34740	PA2311	**Hypothetical, unclassified, unknown**			8.85	-3.04	6.03	3.53	2.96	hypothetical protein
PA14_32890	PA2453	**Hypothetical, unclassified, unknown**		2.79	4.42					hypothetical protein
PA14_06720	PA2485	**Hypothetical, unclassified, unknown**		2.31	2.67	-3.37				hypothetical protein
PA14_30550	PA2594	**Hypothetical, unclassified, unknown**			2.34	-4.22	7.14	4.57	3.80	TauA, ABC-type nitrate/sulfonate/bicarbonate transport systems, periplasmic components [Inorganic ion transport and metabolism].

PA14_46900	PA2630	**Hypothetical, unclassified, unknown**	*ycfD*	3.62	2.89	-4.00			2.63	COG2850, Uncharacterized conserved protein [Function unknown].
PA14_29590	PA2667	**Hypothetical, unclassified, unknown**		2.44	6.90	-3.19	7.18			conserved hypothetical protein
PA14_28500	PA2753	**Hypothetical, unclassified, unknown**		10.13	4.72	-14.22	3.34	4.12	8.93	hypothetical protein
PA14_28490	PA2754	**Hypothetical, unclassified, unknown**		4.12						ElaB, Uncharacterized conserved protein [Function unknown].
PA14_27870	PA2805	**Hypothetical, unclassified, unknown**		2.93	2.10		3.16			hypothetical protein
PA14_25620	PA2971	**Hypothetical, unclassified, unknown**	*yceD*		22.94	-3.27	8.27			COG1399, Predicted metal-binding, possibly nucleic acid-binding protein [General function prediction only].
PA14_25140	PA3009	**Hypothetical, unclassified, unknown**			6.29	-4.02	5.25	4.67	3.13	hypothetical protein
PA14_24880	PA3031	**Hypothetical, unclassified, unknown**				-3.22		3.23	2.21	hypothetical protein
PA14_24300	PA3081	**Hypothetical, unclassified, unknown**		2.15	2.21					conserved hypothetical protein
PA14_21650	PA3276	**Hypothetical, unclassified, unknown**				-3.86	3.62	5.87	2.13	hypothetical protein
PA14_21220	PA3309	**Hypothetical, unclassified, unknown**	*uspK*	4.20	-3.12	-6.71		5.40	10.89	UspA, Universal stress protein UspA and related nucleotide-binding proteins [Signal transduction mechanisms].
PA14_21000	PA3329	**Hypothetical, unclassified, unknown**		2.15	6.39	-12.21	-3.51	5.04	5.13	hypothetical protein
PA14_01610	PA3332	**Hypothetical, unclassified, unknown**		2.67	5.04	-5.26	-3.55			COG3631, Ketosteroid isomerase-related protein [General function prediction only].
PA14_19120	PA3335	**Hypothetical, unclassified, unknown**		2.37	3.51				2.79	hypothetical protein
PA14_20800	PA3345	**Hypothetical, unclassified, unknown**			2.39	-3.86		3.96	2.19	ArcB, FOG: HPt domain [Signal transduction mechanisms].
PA14_19500	PA3449	**Hypothetical, unclassified, unknown**				-6.90	19.03	10.69	6.10	TauA, ABC-type nitrate/sulfonate/bicarbonate transport systems, periplasmic components [Inorganic ion transport and metabolism].
PA14_19205	PA3470	**Hypothetical, unclassified, unknown**			2.49	-7.35				MutT, NTP pyrophosphohydrolases including oxidative damage repair enzymes [DNA replication, recombination, and repair / General function prediction only].
PA14_19170	PA3472	**Hypothetical, unclassified, unknown**			3.04	-2.76				Spr, Cell wall-associated hydrolases (invasion-associated proteins) [Cell envelope biogenesis, outer membrane].

PA14_19020	PA3484	**Hypothetical, unclassified, unknown**		2.15	2.84					hypothetical protein
PA14_68940	PA3496	**Hypothetical, unclassified, unknown**		2.09	3.69		3.52			hypothetical protein
PA14_18370	PA3552	**Hypothetical, unclassified, unknown**	*arnB, pmrH*	2.85	3.13	-13.97	-11.09		-3.09	WecE, Predicted pyridoxal phosphate-dependent enzyme apparently involved in regulation of cell wall biogenesis [Cell envelope biogenesis, outer membrane].
PA14_18100	PA3572	**Hypothetical, unclassified, unknown**		4.00	3.15	-15.67	8.88	6.06	12.53	hypothetical protein
PA14_17580	PA3613	**Hypothetical, unclassified, unknown**		2.40	2.61	-2.74				COG3957, Phosphoketolase [Carbohydrate transport and metabolism].
PA14_34460	PA3662	**Hypothetical, unclassified, unknown**		6.29	4.14		6.42		2.95	hypothetical protein
PA14_16830	PA3675	**Hypothetical, unclassified, unknown**			2.42	-3.28				
PA14_16720	PA3684	**Hypothetical, unclassified, unknown**		3.10	2.75					hypothetical protein
PA14_16180	PA3729	**Hypothetical, unclassified, unknown**		2.15	2.95					COG2268, Uncharacterized protein conserved in bacteria [Function unknown].
PA14_14610	PA3822	**Hypothetical, unclassified, unknown**	*yajC*	2.59	9.26					YajC, Preprotein translocase subunit YajC [Intracellular trafficking and secretion].
PA14_13720	PA3880	**Hypothetical, unclassified, unknown**		2.83	4.20	-14.35		3.11	5.71	COG4273, Uncharacterized conserved protein [Function unknown].
PA14_12370	PA3978	**Hypothetical, unclassified, unknown**		2.36	2.18					COG0790, FOG: TPR repeat, SEL1 subfamily [General function prediction only].
PA14_12360	PA3979	**Hypothetical, unclassified, unknown**			6.13	-3.75	4.91	4.97	3.29	COG4517, Uncharacterized protein conserved in bacteria [Function unknown].
PA14_11250	PA4069	**Hypothetical, unclassified, unknown**			3.51	-3.83		3.22	2.48	RfbD, dTDP-4-dehydrorhamnose reductase [Cell envelope biogenesis, outer membrane].
PA14_56540	PA4348	**Hypothetical, unclassified, unknown**		2.25	5.03	-9.26			3.24	FpaA, Uncharacterized flavoproteins [Energy production and conversion].
PA14_56930	PA4379	**Hypothetical, unclassified, unknown**		2.19	7.75	-6.10	3.27	7.66	3.03	UbiE, Methylase involved in ubiquinone/menaquinone biosynthesis [Coenzyme metabolism].
PA14_57460	PA4421	**Hypothetical, unclassified, unknown**	*yabB*		2.15	-3.92	5.00	3.12	2.56	COG2001, Uncharacterized protein conserved in bacteria [Function unknown].
PA14_57690	PA4441	**Hypothetical, unclassified, unknown**		2.06	2.60					COG3105, Uncharacterized protein conserved in bacteria [Function unknown].

PA14_57850	PA4454	**Hypothetical, unclassified, unknown**	*yrbD*		2.63	-2.34				Ttg2C, ABC-type transport system involved in resistance to organic solvents, periplasmic component [Secondary metabolites biosynthesis, transport, and catabolism].
PA14_60560	PA4577	**Hypothetical, unclassified, unknown**		3.41		-25.84		5.01	9.80	DksA, DnaK suppressor protein [Signal transduction mechanisms].
PA14_71630	PA4610	**Hypothetical, unclassified, unknown**		7.46	3.09	-3.55				hypothetical protein
PA14_61390	PA4639	**Hypothetical, unclassified, unknown**				-4.90		3.07	2.30	hypothetical protein
PA14_61840	PA4674	**Hypothetical, unclassified, unknown**	*vapI*	2.73	2.07		5.04		4.37	VapI, Plasmid maintenance system antidote protein [General function prediction only].
PA14_62170	PA4697	**Hypothetical, unclassified, unknown**		2.42	2.10	-5.18	3.05		2.45	hypothetical protein
PA14_62880	PA4753	**Hypothetical, unclassified, unknown**	*yhbY*	2.11	6.37		5.99			COG1534, Predicted RNA-binding protein containing KH domain, possibly ribosomal protein [Translation, ribosomal structure and biogenesis].
PA14_63110	PA4773	**Hypothetical, unclassified, unknown**		2.11	2.56	-17.18	-4.59	3.19	2.22	SpeD, S-adenosylmethionine decarboxylase [Amino acid transport and metabolism].
PA14_63940	PA4836	**Hypothetical, unclassified, unknown**				-6.06		5.24	3.38	hypothetical protein, Hypothetical protein
PA14_20960	PA4918	**Hypothetical, unclassified, unknown**		2.54	9.01	-5.24	3.51	20.86	5.15	PncA, Amidases related to nicotinamidase [Secondary metabolites biosynthesis, transport, and catabolism].
PA14_68800	PA5208	**Hypothetical, unclassified, unknown**		2.56	4.24	-7.94			4.24	COG1392, Phosphate transport regulator (distant homolog of PhoU) [Inorganic ion transport and metabolism].
PA14_69090	PA5232	**Hypothetical, unclassified, unknown**	*yhiI*	3.77	2.58	-6.99			3.66	EmrA, Multidrug resistance efflux pump [Defense mechanisms].
PA14_62940	PA5475	**Hypothetical, unclassified, unknown**		3.66	3.06	-4.33		4.16	7.46	hypothetical protein
PA14_61500	PA5492	**Hypothetical, unclassified, unknown**	*ysxC, yihA*	2.70	2.29					COG0218, Predicted GTPase [General function prediction only].
PA14_06730	PA5494	**Hypothetical, unclassified, unknown**		6.37	6.03	-5.92			4.03	hypothetical protein
PA14_48790	PA1199	**Membrane proteins**				-3.95		4.80	3.47	Spr, Cell wall-associated hydrolases (invasion-associated proteins) [Cell envelope biogenesis, outer membrane].
PA14_48140	PA1245	**Membrane proteins**	*aprX*		-2.74	-3.62			2.25	hypothetical protein
PA14_45970	PA1429	**Membrane proteins**		6.37	2.26	-7.25			2.29	MgtA, Cation transport ATPase [Inorganic ion transport and metabolism].
PA14_42100	PA1735	**Membrane proteins**		2.15	4.15	-3.50				COG0679, Predicted permeases [General function prediction only].

PA14_32310	PA2501	**Membrane proteins**		3.85	-2.27	-16.45	4.33	4.68	9.80	hypothetical protein
PA14_25470	PA2985	**Membrane proteins**			5.49	-3.21				COG3216, Uncharacterized protein conserved in bacteria [Function unknown].
PA14_21630	PA3278	**Membrane proteins**		9.62	3.47	-15.04	7.93	3.07	7.87	hypothetical protein
PA14_20900	PA3336	**Membrane proteins**		2.38	2.62	-3.79		3.39	2.68	AraJ, Arabinose efflux permease, MFS transporter [Carbohydrate transport and metabolism].
PA14_18060	PA3575	**Membrane proteins**		2.56	4.65	-9.09				CybB, Cytochrome B561 [Energy production and conversion].
PA14_17920	PA3585	**Membrane proteins**	*glpM*		2.02	-3.08				GlpM, Uncharacterized membrane protein required for alginate biosynthesis [General function prediction only].
PA14_14360	PA3839	**Membrane proteins**	*yfbS*	3.40	2.34					CitT, Di- and tricarboxylate transporters [Inorganic ion transport and metabolism].
PA14_47530	PA3877	**Membrane proteins**	*narK1*	27.32	2.35					NarK, Nitrate/nitrite transporter [Inorganic ion transport and metabolism].
PA14_12560	PA3966	**Membrane proteins**		2.17	11.35	-2.15	5.19			hypothetical protein
PA14_11270	PA4067	**Membrane proteins**	*oprG, yciD, ompW*	2.79	4.03	-8.85			3.16	OmpW, Outer membrane protein W [Cell envelope biogenesis, outer membrane].
PA14_09050	PA4243	**Membrane proteins**	*secY, prlA*		2.70	-2.02				SecY, Preprotein translocase subunit SecY [Intracellular trafficking and secretion].
PA14_57030	PA4387	**Membrane proteins**	*fxsA*	3.25		-3.09				FxsA, Protein affecting phage T7 exclusion by the F plasmid [General function prediction only].
PA14_06130	PA4747	**Membrane proteins**	*secG*	2.09	2.02					SecG, Preprotein translocase subunit SecG [Intracellular trafficking and secretion].
PA14_72630	PA5504	**Membrane proteins**			6.13	-3.57	3.65	3.02		AbcD, ABC-type metal ion transport system, permease component [Inorganic ion transport and metabolism].
PA14_73320	PA5561	**Membrane proteins**	*atpI, uncI*		4.39	-3.36	5.53	4.49	2.43	AtpI, F0F1-type ATP synthase, subunit I [Energy production and conversion].
PA14_05340	PA0410	**Motility and Attachment**	*pilI*		2.09	-3.04				CheW, Chemotaxis signal transduction protein [Cell motility and secretion / Signal transduction mechanisms].
PA14_05360	PA0411	**Motility and Attachment**	*pilJ*		3.26	-3.97				Tar, Methyl-accepting chemotaxis protein [Cell motility and secretion / Signal transduction mechanisms].
PA14_37060	PA2128	**Motility and Attachment**	*cupA1*	2.53	2.04	-16.29	7.42	4.11	7.35	FimA, P pilus assembly protein, pilin FimA [Cell motility and secretion / Intracellular trafficking and secretion].
PA14_69940	PA5298	**Nucleotide biosynthesis**	*xpt*		4.13	-4.72				Apt, Adenine/guanine phosphoribosyltransferases and related PRPP-binding proteins [Nucleotide transport and metabolism].
PA14_06360	PA0489	**Putative enzymes**				-6.06	6.33	6.68	5.65	ComFC, Predicted amidophosphoribosyltransferases [General function prediction only].
PA14_07070	PA0545	**Putative enzymes**		11.11	2.89	-8.20		3.10	3.69	COG4097, Predicted ferric reductase [Inorganic ion transport and metabolism].
PA14_53470	PA0836	**Putative enzymes**	*ackA, tdcD*	7.75	2.58	-12.87			2.24	ackA, Acetate kinase [Energy production and conversion].

PA14_48440	PA1225	**Putative enzymes**				-3.52		3.37	2.59	MdaB, Putative NADPH-quinone reductase (modulator of drug activity B) [General function prediction only].
PA14_47340	PA1306	**Putative enzymes**			4.09	-4.65				Hit, Diadenosine tetraphosphate (Ap4A) hydrolase and other HIT family hydrolases [Nucleotide transport and metabolism / Carbohydrate transport and metabolism / General function prediction only].
PA14_46890	PA1344	**Putative enzymes**	*yvaG*		-2.24	-3.16	-4.88			FabG, Dehydrogenases with different specificities (related to short-chain alcohol dehydrogenases) [Secondary metabolites biosynthesis, transport, and catabolism / General function prediction only].
PA14_34750	PA2310	**Putative enzymes**				-7.30	6.98	12.46	8.06	TauD, Probable taurine catabolism dioxygenase [Secondary metabolites biosynthesis, transport, and catabolism].
PA14_21640	PA3277	**Putative enzymes**		2.04	5.08	-9.62			2.38	DltE, Short-chain dehydrogenases of various substrate specificities [General function prediction only].
PA14_17930	PA3328	**Putative enzymes**		2.93	2.49	-5.62			2.76	UbiH, 2-polyprenyl-6-methoxyphenol hydroxylase and related FAD-dependent oxidoreductases [Coenzyme metabolism / Energy production and conversion].
PA14_06740	PA3330	**Putative enzymes**		3.25	14.29	-3.85	-4.52			COG4221, Short-chain alcohol dehydrogenase of unknown specificity [General function prediction only].
PA14_19560	PA3444	**Putative enzymes**	*ssuD*	2.67	3.13		17.69	4.53	3.13	COG2141, Coenzyme F420-dependent N5,N10-methylene tetrahydromethanopterin reductase and related flavin-dependent oxidoreductases [Energy production and conversion].
PA14_51820	PA0963	**Transcription, RNA processing and degradation**	*aspS, syd*		2.53	-2.72				AspS, Aspartyl-tRNA synthetase [Translation, ribosomal structure and biogenesis].
PA14_08710	PA4275	**Transcription, RNA processing and degradation**	*nusG*	2.43	5.95		3.11			NusG, Transcription antiterminator [Transcription].
PA14_33580	PA4944	**Transcription, RNA processing and degradation**	*hfq*	2.56	2.23					conserved hypothetical protein
PA14_06870	PA0527	**Transcriptional regulators**	*dnr*		4.76	-6.71			3.64	transcriptional regulator Dnr
PA14_54430	PA0762	**Transcriptional regulators**	*algU, algT*		4.85	-3.22	5.82	3.96	2.75	RpoE, DNA-directed RNA polymerase specialized sigma subunit, sigma24 homolog [Transcription].
PA14_54400	PA0765	**Transcriptional regulators**	*mucC*			-5.35		3.36	2.16	positive regulator for alginate biosynthesis MucC, regulator of sigma E activity [Signal transduction mechanisms].
PA14_49180	PA1179	**Transcriptional regulators**	*phoP*	2.21			-5.29	-5.85	-4.68	two-component response regulator, OmpR, Response regulators consisting of a CheY-like receiver domain and a winged-helix DNA-binding domain [Signal transduction mechanisms / Transcription].
PA14_32490	PA1603	**Transcriptional regulators**		2.85	2.04	-3.15				MarR, Transcriptional regulators [Transcription].

PA14_41260	PA1799	**Transcriptional regulators**			2.09	-3.22		6.18	2.13	OmpR, Response regulators consisting of a CheY-like receiver domain and a winged-helix DNA-binding domain [Signal transduction mechanisms / Transcription].
PA14_35210	PA2270	**Transcriptional regulators**			3.26	-3.53		3.06	2.23	AcrR, Transcriptional regulator [Transcription].
PA14_26760	PA2885	**Transcriptional regulators**	*atuR*	2.35	4.81	-5.56			2.06	AcrR, Transcriptional regulator [Transcription].
PA14_25180	PA3006	**Transcriptional regulators**	*psrA*	2.32	2.07	-5.10	4.30	3.04	2.28	AcrR, Transcriptional regulator [Transcription].
PA14_20730	PA3351	**Transcriptional regulators**	*flgM*		2.18	-5.24		3.40	2.93	hypothetical protein
PA14_19380	PA3458	**Transcriptional regulators**		7.09	2.88				2.82	MarR, Transcriptional regulators [Transcription].
PA14_18080	PA3574	**Transcriptional regulators**			3.15	-6.25		3.72	3.66	AcrR, Transcriptional regulator [Transcription].
PA14_16280	PA3721	**Transcriptional regulators**		3.66	-8.22		3.47			AcrR, Transcriptional regulator [Transcription].
PA14_12780	PA3948	**Transcriptional regulators**	*rocA1*	2.68	2.23	-3.19				CitB, Response regulator containing a CheY-like receiver domain and an HTH DNA-binding domain [Signal transduction mechanisms / Transcription].
PA14_17490	PA3973	**Transcriptional regulators**		4.37	2.13	-5.03				AcrR, Transcriptional regulator [Transcription].
PA14_60810	PA4596	**Transcriptional regulators**		3.28	3.31	-7.75			2.33	probable transcriptional regulator
PA14_64050	PA4843	**Transcriptional regulators**		2.29	2.39	-4.20			2.36	PleD, Response regulator containing a CheY-like receiver domain and a GGDEF domain [Signal transduction mechanisms].
PA14_00200	PA0019	**Translation, post-translational modification, degradation**	*def*	2.02	3.15	-3.72			2.02	Def, N-formylmethionyl-tRNA deformylase [Translation, ribosomal structure and biogenesis].
PA14_07560	PA0579	**Translation, post-translational modification, degradation**	*rpsU*	2.21	6.94		7.56			RpsU, Ribosomal protein S21 [Translation, ribosomal structure and biogenesis].
PA14_41190	PA1805	**Translation, post-translational modification, degradation**	*ppiD*			-4.93		4.04	2.20	peptidyl-prolyl cis-trans isomerase D
PA14_66750	PA2619	**Translation, post-translational modification, degradation**	*infA*	2.23	2.90	-2.29	3.76		2.27	InfA, Translation initiation factor 1 (IF-1) [Translation, ribosomal structure and biogenesis].
PA14_28720	PA2738	**Translation, post-translational modification, degradation**	*himA*	2.87	2.15	-2.27				HimA, Bacterial nucleoid DNA-binding protein [DNA replication, recombination, and repair].
PA14_27210	PA2851	**Translation, post-translational modification, degradation**	*efp*	2.80	2.58	-2.46				Efp, Translation elongation factor P (EF-P)/translation initiation factor 5A (eIF-5A) [Translation, ribosomal structure and biogenesis].
PA14_25630	PA2970	**Translation, post-translational modification, degradation**	*rpmF*	2.13	2.98		7.95			RpmF, Ribosomal protein L32 [Translation, ribosomal structure and biogenesis].

PA14_21820	PA3262	**Translation, post-translational modification, degradation**				-3.19		3.84	2.82	FkpA, FKBP-type peptidyl-prolyl cis-trans isomerases 1 [Posttranslational modification, protein turnover, chaperones].
PA14_17070	PA3655	**Translation, post-translational modification, degradation**	*tsf*	2.25	3.64					Tsf, Translation elongation factor Ts [Translation, ribosomal structure and biogenesis].
PA14_17060	PA3656	**Translation, post-translational modification, degradation**	*rpsB*		2.24	-2.33	3.07			RpsB, Ribosomal protein S2 [Translation, ribosomal structure and biogenesis].
PA14_16000	PA3742	**Translation, post-translational modification, degradation**	*rplS*	2.80	3.17		5.04			RplS, Ribosomal protein L19 [Translation, ribosomal structure and biogenesis].
PA14_09130	PA4237	**Translation, post-translational modification, degradation**	*rplQ*	2.55	2.54					RplQ, Ribosomal protein L17 [Translation, ribosomal structure and biogenesis].
PA14_20970	PA4245	**Translation, post-translational modification, degradation**	*rpmD*	2.05	4.59	-3.45				RpmD, Ribosomal protein L30/L7E [Translation, ribosomal structure and biogenesis].
PA14_08960	PA4252	**Translation, post-translational modification, degradation**	*rplX*		4.28	-3.23				RplX, Ribosomal protein L24 [Translation, ribosomal structure and biogenesis].
PA14_08750	PA4271	**Translation, post-translational modification, degradation**	*rplL*	2.30	3.20		6.94			RplL, Ribosomal protein L7/L12 [Translation, ribosomal structure and biogenesis].
PA14_58170	PA4482	**Translation, post-translational modification, degradation**	*gatC*	2.04	5.62			-3.23	-2.94	Glu-tRNA(Gln) amidotransferase subunit C
PA14_60450	PA4567	**Translation, post-translational modification, degradation**	*rpmA*		11.47	-2.49	7.27			RpmA, Ribosomal protein L27 [Translation, ribosomal structure and biogenesis].
PA14_71230	PA4934	**Translation, post-translational modification, degradation**	*rpsR*	2.34	11.04		12.22			RpsR, Ribosomal protein S18 [Translation, ribosomal structure and biogenesis].
PA14_66710	PA5049	**Translation, post-translational modification, degradation**	*rpmE*	2.12	12.85	-2.55	16.38			RpmE, Ribosomal protein L31 [Translation, ribosomal structure and biogenesis].
PA14_41970	PA5051	**Translation, post-translational modification, degradation**	*argS*	2.02	2.93	-3.03				ArgS, Arginyl-tRNA synthetase [Translation, ribosomal structure and biogenesis].
PA14_70190	PA5316	**Translation, post-translational modification, degradation**	*rpmB*		17.30	-2.75	13.54			RpmB, Ribosomal protein L28 [Translation, ribosomal structure and biogenesis].

PA14_73420	PA5569	**Translation, post-translational modification, degradation**	*rnpA*		2.72	-3.65				RnpA, RNase P protein component [Translation, ribosomal structure and biogenesis].
	Pae_tR	**Translation, post-translational modification, degradation**		2.49	2.03		5.50	6.39	4.41	tRNA_Isoleucine, 723696-723772 (+) strand
PA14_45110	PA1493	**Transport of small molecules**	*cysP*			-7.30		4.90	4.12	Sbp, ABC-type sulfate transport system, periplasmic component [Inorganic ion transport and metabolism].
PA14_34410	PA2339	**Transport of small molecules**	*mtlF*	2.21	9.88				-2.07	UgpA, ABC-type sugar transport systems, permease components [Carbohydrate transport and metabolism].
PA14_03080	PA2505	**Transport of small molecules**	*opdT, oprD3*	2.11	2.56					hypothetical protein
PA14_24790	PA3038	**Transport of small molecules**	*opdQ*	2.51	5.99	-3.26				hypothetical protein
PA14_18670	PA3531	**Transport of small molecules**	*bfrB*	7.81	10.59	-14.04	8.58	4.78	5.88	bacterioferritin, bfrB (bacterioferritin)
PA14_12920	PA3938	**Transport of small molecules**	*tauA*		11.56	-5.43	8.18	11.54	4.26	TauA, ABC-type taurine transport system, periplasmic component [Inorganic ion transport and metabolism].
PA14_09160	PA4235	**Transport of small molecules**	*bfrA*	2.02	2.21	-5.68			2.49	bacterioferritin
PA14_57960	PA4464	**Transport of small molecules**	*ptsN*	2.90	2.08					PtsN, Phosphotransferase system mannitol/fructose-specific IIA domain (Ntr-type) [Carbohydrate transport and metabolism / Signal transduction mechanisms].
PA14_58570	PA4514	**Transport of small molecules**	*piuA*	2.22	2.22	2.01	-3.58		-2.38	probable outer membrane receptor for iron transport, piuA.
PA14_05320	PA0408	**Two-component regulatory systems**	*pilG*	2.90	6.99	-8.93	3.78	3.70	3.97	twitching motility protein PilG
PA14_05330	PA0409	**Two-component regulatory systems**	*pilH*	2.71	3.00	-5.71			2.13	twitching motility protein PilH
PA14_58320	PA4494	**Two-component regulatory systems**			2.74	-3.34				BaeS, Signal transduction histidine kinase [Signal transduction mechanisms].
