# Supplementary material for: Homeostatic Interplay between Bacterial Cell-Cell Signaling and Iron in Virulence
Source: PLoS Pathog. 2010 Mar 12;6(3):e1000810. doi: 10.1371/journal.ppat.1000810 (PMC2837411; doi:10.1371/journal.ppat.1000810)
Supplement: Table S4 — Strains, plasmids and primers. The P. aeruginosa strains, plasmids and primers that were used in this study. (0.07 MB DOC) [file ppat.1000810.s010.doc]

**Strain description and genotype Source**

***P. aeruginosa* PA14 strains**

PA14 Clinical isolate UCBPP-PA14 [1]

*mvfR**^-^* A nonsense point mutation [2]

*pqsE^-^* *pqsE* non-polar deletion mutant [3]

*pqsA^-^* Non polar deletion of *pqsA* [3]

*pqsH^-^ aacC1* cassette inserted into *pqsH* [4]

Gm^r^, Km^r^

*pqsA^-^pqsH^-^ pqsA^-^* containing an *aacC1* cassette [4]

inserted into *pqsH*; Gm^r^, Km^r^

*phzC1^-^phzC2^-^* Double deletion of the *c* genes This study

In both *phz* operons

*phzM^-^* Non polar deletion This study

*rhlR^-^ rhlR*::Gm^r^ Lab collection

AA^-^ Δ*phnAB*, *trpE*::Gm^r^, *kynBU*::km^r^ [5]

***P. aeruginosa* PAO1 strain**

PAO1 [6]

***E. coli***

JM109 F–(*traD36 proAB*+*lacI*q*lacZ* D*M* Lab

*endA1 reacA1 hsdR17*(rK– mK+) collection

*supE44 thi-1 gyrA96 relA1* D

(*lac-proAB*)

S17-1 Smr Tpr*mod*+*res thi pro recA hsdR17* Simon *et al*. (1983)

integrated plasmid RP4-TC::Mu-

Km::Tn*7* into genome

**Plasmids**

**Name description Source**

pDN19 Expression vector; Tet^r^ [7]

pDN19*pqsE* pDN19 p*lac*-*pqsE;* Tet^r^ This study

pDN18 Expression vector; Tet^r^ [7]

pDN18*mvfR* pDN18 p*lac-mvfR;* Tet^r^ [3]

pAC37 *pqsA*-GFP (ASV) [8]

pED1 *mexG’ –lacZ;* Crb^r^ [9]

pED3 *phzA2 –lacZ;* Crb^r^ This study

pECP60 *rhlA*¢*–lacZ* translational fusion, Crb^r^ [10]

pME3826 *hcn*–*lacZ* , Tet^r^ [11]

pUCP-A2G2 Clone of *phzA2B2C2D2E2F2G2;* Tet^r^ [12]

pUCP-MS Clone of *phzM* and *phzS*; Tet^r^ [12]

pGYMC*rhlR* Clone of rhlR on pUCP20 [13]

**Primers**

*pqsE* cloning

GX119 TTGCCAAGCTTGAGGCTTTCGGCTCCCGG

GX120 AATCCTCTAGATCAGT CCAGAGGCAGCGCCTG

*RT-PCR, pqsA*

q_pqsA_F ACCGTGATCAATCCCAAGTC

q_pqsA_R GAGAAATCGTCGAGCAAAGG

*RT-PCR, pqsE*

q_pqsE_F ATGATGACCTGTGCCTGTTG

q_pqsE_R GTCGTAGTGCTTGTGGGTGA

*RT-PCR, rpoD*

PA0576F CTGATCCAGGAAGGCAACAT

PA0576R TGAGCTTGTTGATCGTCTCG

**References**

1. Rahme LG, Stevens EJ, Wolfort SF, Shao J, Tompkins RG, et al. (1995) Common virulence factors for bacterial pathogenicity in plants and animals. Science 268: 1899-1902.

2. Cao H, Krishnan G, Goumnerov B, Tsongalis J, Tompkins R, et al. (2001) A quorum sensing-associated virulence gene of Pseudomonas aeruginosa encodes a LysR-like transcription regulator with a unique self-regulatory mechanism. Proc Natl Acad Sci U S A 98: 14613-14618.

3. Deziel E, Lepine F, Milot S, He J, Mindrinos MN, et al. (2004) Analysis of Pseudomonas aeruginosa 4-hydroxy-2-alkylquinolines (HAQs) reveals a role for 4-hydroxy-2-heptylquinoline in cell-to-cell communication. Proc Natl Acad Sci U S A 101: 1339-1344.

4. Xiao G, Deziel E, He J, Lepine F, Lesic B, et al. (2006) MvfR, a key Pseudomonas aeruginosa pathogenicity LTTR-class regulatory protein, has dual ligands. Mol Microbiol 62: 1689-1699.

5. Lesic B, Rahme LG (2008) Use of the lambda Red recombinase system to rapidly generate mutants in Pseudomonas aeruginosa. BMC Mol Biol 9: 20.

6. Holloway BW, Morgan AF (1986) Genome organization in Pseudomonas. Annu Rev Microbiol 40: 79-105.

7. Nunn D, Bergman S, Lory S (1990) Products of three accessory genes, pilB, pilC, and pilD, are required for biogenesis of Pseudomonas aeruginosa pili. J Bacteriol 172: 2911-2919.

8. Yang L, Barken KB, Skindersoe ME, Christensen AB, Givskov M, et al. (2007) Effects of iron on DNA release and biofilm development by Pseudomonas aeruginosa. Microbiology 153: 1318-1328.

9. Deziel E, Gopalan S, Tampakaki AP, Lepine F, Padfield KE, et al. (2005) The contribution of MvfR to Pseudomonas aeruginosa pathogenesis and quorum sensing circuitry regulation: multiple quorum sensing-regulated genes are modulated without affecting lasRI, rhlRI or the production of N-acyl-L-homoserine lactones. Mol Microbiol 55: 998-1014.

10. Pesci EC, Pearson JP, Seed PC, Iglewski BH (1997) Regulation of las and rhl quorum sensing in Pseudomonas aeruginosa. J Bacteriol 179: 3127-3132.

11. Blumer C, Heeb S, Pessi G, Haas D (1999) Global GacA-steered control of cyanide and exoprotease production in Pseudomonas fluorescens involves specific ribosome binding sites. Proc Natl Acad Sci U S A 96: 14073-14078.

12. Mavrodi DV, Blankenfeldt W, Thomashow LS (2006) Phenazine compounds in fluorescent Pseudomonas spp. biosynthesis and regulation. Annu Rev Phytopathol 44: 417-445.

13. Medina G, Juarez K, Valderrama B, Soberon-Chavez G (2003) Mechanism of Pseudomonas aeruginosa RhlR transcriptional regulation of the rhlAB promoter. J Bacteriol 185: 5976-5983.
